# Measurement of the Effect of Accelerated Aging on the Aromatic Compounds of Gewürztraminer and Teroldego Wines, Using a SPE-GC-MS/MS Protocol

**DOI:** 10.3390/metabo12020180

**Published:** 2022-02-15

**Authors:** Silvia Carlin, Cesare Lotti, Ludovica Correggi, Fulvio Mattivi, Panagiotis Arapitsas, Urška Vrhovšek

**Affiliations:** 1 Department of Food Quality and Nutrition, Edmund Mach Foundation, Research and Innovation Centre, Via Edmund Mach 1, 38010 San Michele all’Adige, TN, Italy; cesare.lotti@fmach.it (C.L.); fulvio.mattivi@unitn.it (F.M.); panagiotis.arapitsas@fmach.it (P.A.); urska.vrhovsek@fmach.it (U.V.); 2 Department of Food and Drug, University of Parma, Area Parco delle Scienze 27/A, 43124 Parma, PR, Italy; ludovica.correggi@hotmail.it; 3 Department of Cellular Computational and Integrative Biology, University of Trento, Via Sommarive 9, 38123 Povo, TN, Italy; 4 Department of Wine, Vine and Beverage Sciences, School of Food Science, University of West Attica, Ag. Spyridonos Str., Egaleo, 12243 Athens, Greece

**Keywords:** VOCs (volatile organic compounds), terpenes, norisoprenoids, Vitis, GC-MS-MS (gas chromatography-tandem mass spectrometry), accelerate aging

## Abstract

Knowing in detail how the white and red wine aroma compounds behave under various storage conditions and especially at high temperature is important in order to understand the changes occurring to their sensorial character during the shelf life. The initial aim of this work was to develop and validate a fast, modern, robust, and comprehensive protocol for the quantification of 64 primary, secondary, and tertiary volatile compounds by using solid-phase extraction (SPE) cartridges in sample preparation and fast GC-MS/MS (gas chromatography-tandem mass spectrometry assay) in analysis. The protocol was applied to a study of the behavior of seven Gewürztraminer and seven Teroldego wines stored in anoxia at 50 °C for 2.5 and 5 weeks. The results demonstrated a sharp decrease of the main linear terpenes linalool, geraniol, and nerol and the consequent increase of the cyclic ones, such as α-terpineol and 1,8-cineole; the increase of the C13-norisoprenoids 1,1,6,-trimethyl-1,2-dihydronapthalene (TDN), and β-damascenone and the C10 norisoprenoid safranal; the hydrolysis of acetates and linear esters; and the increase of some branched-chain esters. In red wines, a moderate increase was observed for some lactones. Some unwanted compounds, such as 2-aminoacetophenone (2-AAP), showed a notable increase in some Gewürztraminer wines, exceeding the olfactory threshold.

## 1. Introduction

The analysis of volatile compounds in wine is an informative tool for characterizing the different cultivars and wine styles and for studying their sensory properties and the dynamic evolution of their composition during maturation and aging. Indeed, we know that wine is one of the beverages that can often evolve and improve during the maturation phase between the fermentation and the bottling as well as during the aging in bottle if this is done in optimal conditions [1,2].

The complete analysis of the wine aroma is, however, complex, time consuming, and expensive. The concentration of the key compounds contributing to the aroma of wines has an extremely wide range of concentration (ng-mg/L) and equally diverse chemical characteristics that sometimes require specific and selective detection methods [3]. The main classes of compounds that impact the fruity and flowery aroma of wines and that modify it over time are the terpenes and norisoprenoids (i.e., varietal or primary aroma compounds) and various esters and alcohols that are formed during fermentation (i.e., secondary aroma compounds). During the development of the wine aroma, compounds that were bound to the precursors can be released, and various chemical rearrangement reactions can take place, delivering the tertiary aroma compounds [4]. Ideally, each wine should be consumed neither before nor after its optimal time. The winemakers are in control of the maturation phase at the winery, usually in barrels or tanks, while the aging phase after bottling is lengthy and difficult to predict. Understanding how wine will evolve over time is a very important aspect for producers to distribute only wines with the best potential for aging. One of the ways used in the past to estimate the aromatic potential depending on the evolution of precursors was to perform chemical or enzymatic hydrolysis to quantify the aroma precursors. Unfortunately, most of these techniques are not always able to simulate the reactions that occur in wine because they use extreme conditions of pH, temperature, and/or concentrations of enzymes with very high α-glucosidase activity. A possible alternative to analyze the potential of wines over time is to simulate the accelerated aging by playing on temperatures [5,6].

Equally important is simplifying and reducing the time required for the extraction and analysis of the most important classes of wine aromas, modifying previously validated protocols. One of the most common methods used for sample preparation/cleaning up and concentrating the volatile compounds is solid-phase extraction (SPE), which can handle a wide range of chemical classes and concentrations. Over the years, this technique has evolved; in 1985, Gunata et al. [7] began to use it to analyze both the free and bound fractions with glass column filled with Amberlite XAD-2 resin, then moved to cartridges already filled with stationary phase [8], and so on, trying to reduce the amount of stationary phase and the amount of solvents [9,10]. However, there is still room for improvement, especially to save time and use fewer amounts of (hazardous) chemicals. Such protocols can be further improved by decreasing the analysis time, which often requires more than an hour when conventional GC-MS (gas chromatography-mass spectrometry) instruments are used [4,11,12]. Modern instruments, such as fast GC-MS/MS, could help the analyst/researcher to analyze more samples at the same time and gain in selectivity and sensitivity.

The purpose of this work was to develop and validate a modern, fast, and comprehensive analytical method able to identify and quantify the majority of wine aroma compounds and to address the need to monitor them in wine science studies. The detailed aims included the individuation of a cartridge able to reduce the quantity of organic solvent necessary to elute and completely eliminate the concentration step by improving previous time consuming, expensive, and complicated multistep protocols [13]. Additionally, the study intended to find a targeted, sensitive, fast, and high-throughput GC-MS/MS method. Using the Intuvo GC system (Agilent) with its compact, planar design column and taking advantage of the rapid heating and cooling capability, it is possible to work efficiently and quickly with faster and more reproducible cycle times. Coupling this separation with a triple quadrupole mass spectrometer also allows a high selectivity and sensitivity.

The final aim was to apply the protocol to a proof-of-concept pilot study and explore how wine storage at high temperature and in anoxic conditions influences the aromatic profile of white and red wines.

## 2. Results and Discussion

### 2.1. Extraction and GC-MS/MS Method Optimization

**Figure 1:**
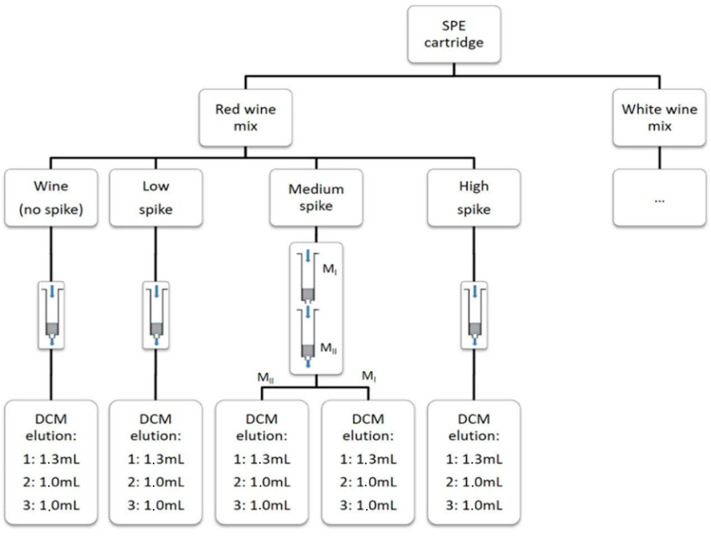
The experimental design used to develop the SPE sample preparation. The same process was used for each cartridge type. Medium spike level (M_I_ and M_II_) cartridges were then separated eluted.

The performances of 3 different cartridges with 200 mg of stationary phase (Bond Elut ENV (Agilent Technologies, Santa Clara, CA, USA), Isolute^®^ ENV+ (Biotage, Uppsala, Sweden) and LiChrolut^®^ EN (Merk, Darmstadt, Germany)) were evaluated. To evaluate the effectiveness of the stationary phase of the cartridge in retaining all the compounds of the wine, we created wine mixes at 3 different levels of concentration (low, medium, and high). Then, 50 mL of this wine mix were loaded into each column, and after that, a first dichloromethane (DCM) fraction of 1.3 mL was eluted in order to estimate if this first fraction was able to elute all the free aroma compounds; two other 1 mL DCM fractions were eluted, and all these DCM fractions were then separately collected and analyzed. For the medium concentration wine mix, 2 cartridges were used, superimposing them on top of each other so that all the wine samples, after passing through the first cartridge (M_I_), also passed through the cartridge below (M_II_); then, each cartridge was separately eluted with the 3 separate DCM fractions (1.3 mL, 1 mL, 1 mL) in order to verify if the stationary phase of the first cartridge was sufficient to retain all the compounds or some of these passed into the cartridge placed below. We analyzed a total of 90 DCM fractions (Figure 1).

The results obtained from the various extractions showed that in all the 3 cartridges, part of the first 1.3 mL DCM fraction remained trapped into the resin. However, almost half remained in the Bond Elut ENV cartridge, and some water was also retained. These cartridges were also found to be less efficient for the extraction of alcohols and some esters, and for all these reasons, the Bond Elut ENV was excluded (Figure S1, Table S1).

The other two cartridges, Isolute^®^ ENV+ and LiChrolut^®^ EN, had very similar performance. However, while the experiment was ongoing, we learned that the latter will soon be removed from the market, so we decided to further validate the method with the Isolute^®^ ENV+ cartridges. Considering that in these cartridges, too, a small amount of compounds was found in the second DCM fraction, it was decided to elute with 2 mL instead of 1.3 mL of DCM. To evaluate the repeatability of the method, technical replicates were made within one day (intraday) and between-day (interday) using both white and red wine mixes. Repeatability (Supplementary Table S2) of the extraction resulted in a CV% below 10% for most compounds (*n* = 70). For two compounds, the CV% gave values between 10% and 20%, which were still acceptable. Only 2 compounds, acetoin (intraday and interday) and phenylacetaldehyde (interday), in the red wine samples, gave values over 20% and therefore were excluded from the method. For white wine, all the CV% values were below 16%. R2 was in a range from 0.9907 to 0.9999 for all compounds and indicated good fit and linearity for the calibration curves in relation to the scope of the method.

Most of the compounds (*n* = 48) gave optimal recovery values between 80–120%, and 13 compounds gave a recovery between 60–80%. Only a dozen compounds in both red and white wines gave values <50%; these were mostly high polar compounds, which are unable to bind to the non-polar stationary phase of styrene divinylbenzene, or acid compounds, for which the pH of the matrix should be changed, with the risk of losing other compounds of interest. For some compounds present in large quantities, such as ethyl esters, diethyl succinate, octanoic acid, decanoic acid, and benzyl alcohol, we tried to increase the split ratio in the GC injector from 1:10 to 1:150, obtaining better results. Both in red and white wines, the recovery values of menthalactone thus improved, probably due to a reduction of the baseline in the chromatogram. However, considering that the 1:10 split ratio is better for the vast majority of compounds, it was decided to use that injection condition and to inject with the highest splitting ratio (1:150) only to quantify the compounds present at higher concentration (Table S2). The limits of quantification (LOQ) for all compounds were suitable for their quantification both in red and white wines. The linearity for the major compounds could be increased using the highest splitting ratio (1:150). The chromatographic run of only 16 min allows a high production capacity. The extraction method, together with the fast GC-MS/MS analysis, made it possible to significantly reduce the use of the DCM solvent, with advantages in terms of operator safety as well as time, avoiding further concentrations of the extracts and allowing the quantification of 64 compounds. All the validation parameters are reported in Table 1 and Table S2.

This validated method was used to monitor the behavior of volatile compounds in Gewürztraminer wines and in autochthonous red wines of the Teroldego variety during an accelerated aging period, and the results are reported in Table 2 and Table 3.

### 2.2. Accelerated Aging

A small experiment was carried out to evaluate the repeatability of the accelerated aging method. Five technical replicates of two different Gewürztraminer wines were placed for 4 days at 40 °C and then analyzed. The results are shown in Table S3 and show, for all compounds, the CV% is below 16%. In consideration of these results, it was decided to conduct the experiment using seven biological replicas of commercial wines for both Gewürztraminer and Teroldego. The wines were analyzed at time 0 (t0) and after 2.5 (t1) and 5 weeks (t2) at 50 °C following the method proposed by Ferreira [14]. The oxygen content was also measured during the first week of storage. The dissolved oxygen content at time zero in the different samples was very variable, also depending on the type of cap used, but always under 578 ppb for red wines and under 604 ppb in white wines. It was also seen that already after 2–3 days, the concentration was very low, under 50 ppb for all the samples.

### 2.3. Gewürztraminer Wine

This aromatic variety with scent of rose petals, cloves, lychees, and other tropical fruits is a variety widely cultivated in the Trentino Alto Adige region located in northern Italy, especially in the area of Tramin, and it has long been studied by various researchers to try to understand what the most characterizing components are [15,16,17].

**Figure 2:**
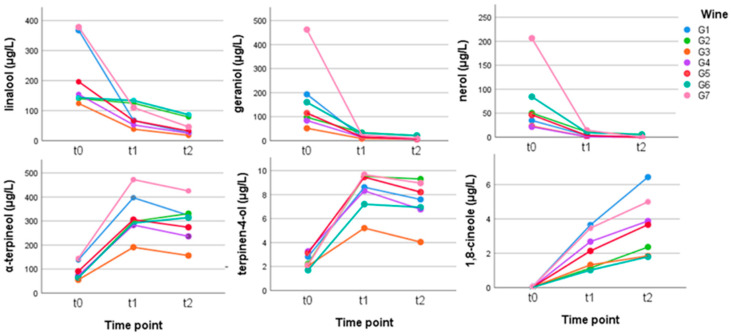
Behavior of the main terpenes and of some compounds derived from them in the Gewürztraminer wines stored in anoxia at time zero (t0) and for 2.5 (t1) and 5 (t2) weeks at 50 °C. (Tukey’s HSD demonstrated a statistically significant difference between t0 and the t1–t2 for all the six compounds (Table S4)).

Terpenes and monoterpenols, particularly geraniol, *cis* rose oxide, citronellol, and linalool, are responsible for the characteristic floral aroma in the *Vitis vinifera* cv. Gewürztraminer grapes and wines [16]. During wine processing and aging, many acid-catalyzed rearrangements take place, mainly with an increase in cyclic forms or hydroxylated derivatives, and this involves changes in concentration and the formation of new volatile compounds that were not present in the grapes or in young wines. Usually the open-chain monoterpene alcohols have a lower perception threshold than their cyclic equivalents, and this accounts for the reduction in the typical floral aroma during storage or aging (reference). The data analysis of the measurements demonstrated a substantial decrease of the mean values by 79% for linalool, by 92% for nerol, by 93% for geraniol, and by 78% for citronellol (Figure 2). The one-way ANOVA analysis pointed out that this change was statistically significant already after 2.5 weeks of accelerated aging (Table S4). On the contrary, the mean values of cyclic α-terpineol and terpinen 4-ol showed a statistically significant increment the first 2.5 weeks (from t0 to t1), correspondingly from 90.14 μg/L to 319.94 μg/L and from 2.47 μg/L to 8.28 μg/L (Figure 2 and Table S4). It is interesting to note that the 1,8-cineole content increased considerably, going from 0.04 μg/L to 3.57 μg/L, and in the two wines with linalool content above 300 μg/L, which also had a higher concentration of α-terpineol, this compound was present in an amount of more than 5 μg/L at the end of the 5 weeks; this supports the theory that this compound may form from linalool cyclization reactions (Figure 2) [4]. Furthermore, the increase of 1,8-cineole was statistically significant after 2.5 weeks if we consider the mean value of the seven biological replicates (Table S4). Previous studies showed that α-terpineol can be formed from limonene under acidic conditions but could also derive from the cyclization of geraniol, nerol, and linalool; after that, α-terpineol can be transformed directly into 1,8-cineole or into 1,8-terpine and this latter compound to 1,8-cineole [4]. 1,8-cineole, with a eucalyptus odor and a very low threshold of around 2 μg/L [18,19], could contribute to the wine aging aroma. Pyranic oxides of linalool were among the compounds that increased during the study, probably due to hydrolysis from the bound forms (+1322%). During fermentation and aging, the aglycones should be freed from precursors; however, in this experiment, probably due to the too-high temperature, no initial increase was observed, while there was a decrease. It is assumed that they undergo rearrangement towards more stable cyclic forms very quickly after the hydrolysis.

**Figure 3:**
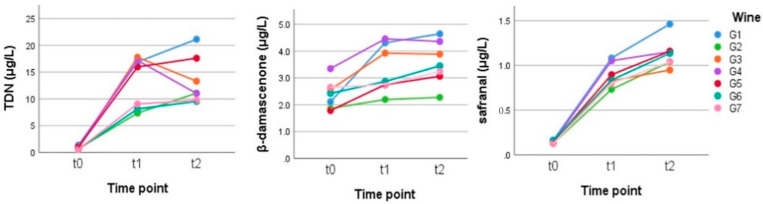
Behavior of the main norisoprenoids in the Gewürztraminer wines stored in anoxia at time zero (t0) and for 2.5 (t1) and 5 (t2) weeks at 50 °C. (Tukey’s HSD: t0 a, t1 b, and t2 b for TDN; t0 a, t1 ab, and t2 b for β-damascenone; and t0 a, t1 b, and t2 c for safranal (Table S4).

Norisoprenoids are among the most important evolutionary wine aroma compounds; they can be formed by direct degradation of carotenoids, such as β-carotene and neoxanthin, or they can be stored as glycoconjugates, which can then release their volatile aglycone during fermentation or aging via enzymatic and acid hydrolysis processes. The carotenoid content in grapes, the fermentation process and the wine storage conditions are factors that greatly influence the evolutionary profile of the wine [20]. One of the most important norisoprenoids is certainly β-damascenone that, with its very low threshold (50 ng/L), manages to contribute to the aroma of the wine both directly and indirectly as an enhancer of the fruity note [21]. During the experiment (Figure 3), the mean content in this compound increased slightly but was statistically significant after 2.5 weeks, from 2.39 μg/L to 3.32 μg/L. The TDN mean content increased statistically significant already after 2.5 weeks, too, but was considerably more by up to 12 times, from 0.87 to 13.19 μg/L (Figure 3 and Table S4). Such a behavior is in accordance with the literature since the production of TDN in wine is promoted by the heating [22,23,24].

Another compound that increased greatly with heating was safranal, (2,6,6-trimethyl-1,3-cyclohexadiene-1-carboxaldehyde), which went from 0.14 μg/L at t0 to 1.13 μg/L at t2. This increase was statistically significant both between t0 and t1, and t1 and t2 (Table S4). Safranal is the main aroma component of saffron; in wine, it exists in free form [25], but given its considerable increase after heating and also in light of the observed increase of its concentration in reserve sparkling wines [25], it is possible to confirm the presence of some precursors. In saffron, the main monoterpene glycoside precursor of safranal is picrocrocin [26]. β-glucosidase action, thermal treatment, or alkaline-acid hydrolysis on picrocrocin liberate the aglycone directly or enzymatically, with the formation of 4-hydroxy-2,6,6-trimethyl-1-cyclohexene-1-carboxaldehyde (HTCC, C_10_H_16_O_2_), which is transformed to safranal by dehydration during the drying process of the plant material [27]. It was also reported that crocetin dialdehyde could be oxidized and esterified to generate crocetin esters, which could also be a safranal precursor after an enzymatic or thermal treatment [28]. In our samples, however, in the untreated wine samples (t0), we did not find any picrocrocin peak, which can therefore lead to the hypothesis that safranal is formed starting from some other precursor or by the rearrangement of some other molecules.

**Figure 4:**
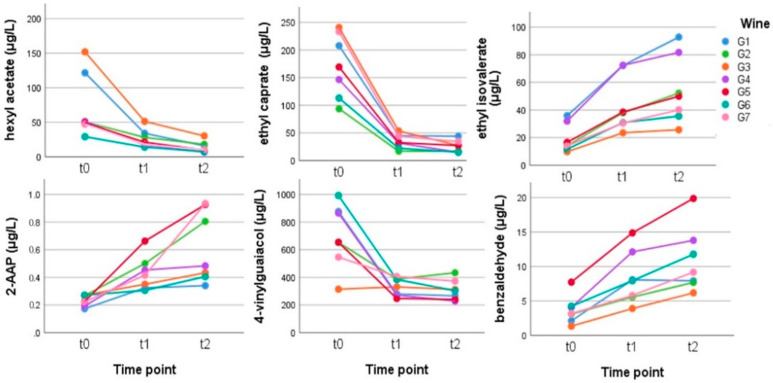
Behavior of some esters and benzenoid compounds in the Gewürztraminer wines stored in anoxia at time zero (t0) and for 2.5 (t1) and 5 (t2) weeks at 50 °C. (Tukey’s HSD: t0 a, t1 b, and t2 b for all except 2-AAP, which was t0 a, t1 ab, and t2 b).

Esters and Acetates: the behavior of acetates and linear ethyl esters, as widely demonstrated in literature, includes a rapid decrease during aging, especially if the wine is not stored in suitable conditions [29]. Figure 4 shows that hexyl acetate decreased rather quickly, going from an average value of 71.55 μg/L to 26.30 μg/L after 2.5 weeks and to 14.09 μg/L at the end of the experiment. Isobutyl acetate showed a similar trend, from the initial concentration of 46.15 μg/L to the final one of 24.85 μg/L (Table S4). Octanoic and decanoic ethyl esters also decreased by 63% and 85%, respectively. In the family of fruity esters, the ethyl esters of the branched acids followed a completely different aging pattern compared to linear ethyl esters and acetates. The levels of these esters progressively increased during aging in a statistically significant way (Table S4). Ethyl 2-methylbutyrate and ethyl 3-methylbutanoate (ethyl isovalerate) exhibited the opposite behavior and increased by +168% and 182%, respectively. Ethyl 2-hydroxy-4-methylpentanoate (ethyl leucate) was identified for the first time in red and white table wines as a compound directly associated with a “fresh blackberry” aroma [30]. This ester increased by +96% between the beginning and the end of the experiment. Aging would seem to favor an increase in the overall concentration of ethyl leucate [9] since the acid-ester equilibrium was the most effective in generating the branched fatty acid ethyl esters from their corresponding acids during wine aging [31]. Diethyl succinate (+60%), as already reported for the esters of diprotic acids, increased during aging and were sometimes used as aging markers [32,33].

Phenols: a very important compound for the spicy note of Gewürztraminer is 4-vinylguaiacol, which brings clove notes and is often present in quantities much higher than its olfactory threshold (40 μg/L) [34]. The behavior of this compound during aging is well known: it tends to decrease rapidly, with the half-life of vinylphenol in white wines being approximately 6 months at 16–18 °C [35]. It was found that the main degradation product of 4-vinylguaiacol in beer was apocynol (4-(1-hydroxyethyl)-2-methoxyphenol) [36], while another possibility is that 4-vinylguaiacol could react with ethanol to form ethoxyethyl phenols, as observed in some wines [37]. In our case, we observed a statistically significant loss of 56% of 4-vinylguaiacol in 5 weeks at 50 °C (Table S4).

Other important benzenoids: methyl salicylate is an organic ester naturally produced by many plants, particularly wintergreens, and also present in wine, sometimes in quite high quantities, such as in the Verdicchio and Lugana varieties [38,39]. It was demonstrated that it could be present in both free and bound form (MeSA glycosides). In small quantities, it is also present in Gewürztraminer, and during the experiment, the content increased a little due to hydrolysis by the glycosides although it remained very far from the olfactory threshold (50 μg/L) and is not statistically significant. 2-Aminoacetophenone (2-AAP) is a known compound since it is considered the main cause of the so-called untypical “aging off-flavor” (UTA) in *Vitis vinifera* wines. According to the literature, the formation of 2-AAP is caused by the oxidative degradation of the phytohormone indole-3-acetic acid (IAA) after fermentation. 2-AAP was identified as the character impact compound, with an odor threshold of about 1 μg/L in wine by [40,41]. In this experiment (Figure 4), the initial value of 0.23–0.27 μg/L of this compound was very similar for all the wines, while at the end of the 5 weeks, in three wines, it had increased to very close (0.81–0.93 μg/L) to the sensory threshold.

### 2.4. Teroldego Wines

Teroldego is a red autochthonous variety from the Trentino-Alto Adige region in northern Italy, and despite their dark color, Teroldego grapes produce wines that have bright fruity notes.

**Figure 5:**
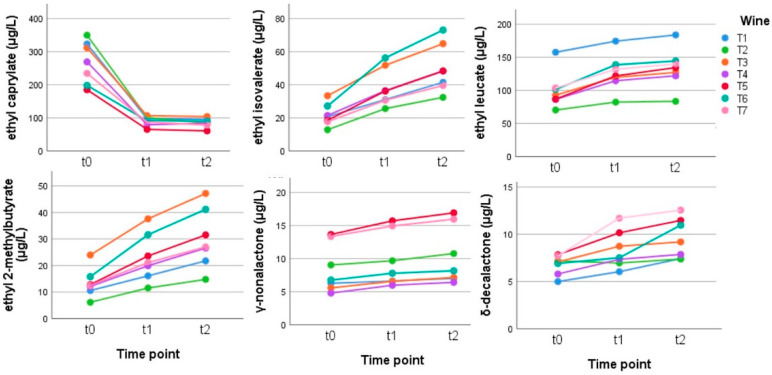
Behavior of some esters and lactones in the Teroldego wines stored in anoxia at time zero (t0) and for 2.5 (t1) and 5 (t2) weeks at 50 °C. (Tukey’s HSD: ethyl caprylate and ethyl isovalerate: t0 a, t1 b, and t2 b for ethyl 2-methylbutyrate and δ-decalactone: t0 a, t1 ab, and t2 b (Table S5)).

Esters and acetates: the same behavior was seen also for Teroldego wines, with acetates, such as isopentyl acetate and hexyl acetate, and linear esters ethyl hexanoate, octanoate, and decanoate that decreased (−24%, −68%, −91%) with aging and branched esters, such as ethyl 2-methylbutyrate, ethyl isovalerate, and ethyl leucate, which increased (+123%, +129%, +34%); these values refer to the mean of seven biological replicates, and the statistical analysis can be found in Table S5. Diethyl succinate also increased (+9%). The amounts of lactones, in particular of γ-nonalactone and δ-decalactones, slightly increased (+21%, +40%) (Figure 5).

**Figure 6:**
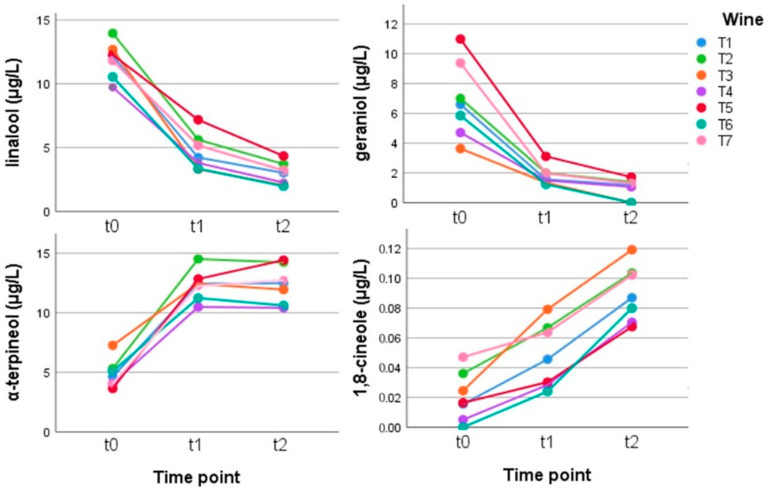
Behavior of the main terpenes and of some compounds derived from them in the Teroldego wines stored in anoxia at time zero (t0) and for 2.5 (t1) and 5 (t2) weeks at 50 °C. (Tukey’s HSD: linalool and 1,8-cineole: t0 a, t1 b, and t2 c and for geraniol and α-terpineol: t0 a, t1 b, and t2 b (Table S5)).

Terpenes: the content in terpene compounds in Teroldego was quite low, but their behavior was very similar to that seen in Gewürztraminer, with linalool decreasing (–75%) and α-terpineol and 1,8-cineole increasing during aging (+155%, +273%) (Figure 6).

**Figure 7:**
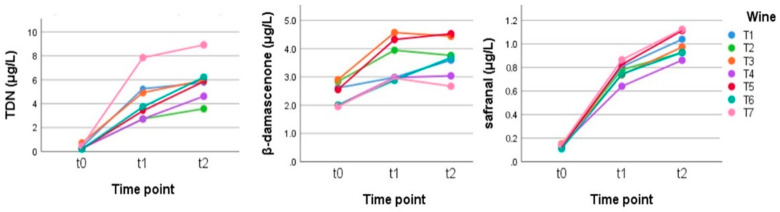
Behavior of the main norisoprenoids in the Teroldego wines stored in anoxia at time zero (t0) and for 2.5 (t1) and 5 (t2) weeks at 50 °C. (Tukey’s HSD: TDN and β-damascenone: t0 a, t1 b, and t2b and for safranal: t0 a, t1 b, and t2 c (Table S5)).

Norisoprenoids: In this red wine as well, TDN, β-damascenone, and safranal increased a great deal during the experiment due to hydrolysis/rearrangement from its precursors (Figure 7).

2-AAP: in Teroldego wines, the content of 2-AAP did not increase during the experiment; in fact, the aging off-flavor (UTA) has not yet been detected in red wines, and red wines spiked with the precursor indole-3-acetic acid before fermentation did not show any significant formation of 2-AAP [42,43]. In fact, red wines are far richer in polyphenols than white wines, which are able to protect wine from oxidation, including the reactions driving to the release of 2-AAP.

## 3. Materials and Methods

### 3.1. Chemicals and Reagents

All standards used in this study are listed in Table S2. Ethanol 99.8%, n-heptanol 99.9%, dichloromethane 99.8%, and methanol for HPLC 99.9% were purchased from Sigma-Aldrich (St. Luis, MO, USA); 3 cartridges with 200 mg of stationary phase based on styrene divinylbenzene were tested for solid-phase extraction (SPE): LiChrolut^®^ EN (Merk, Darmstadt, Germany), Isolute^®^ ENV+ (Biotage, Uppsala, Sweden), and Bond Elut ENV (Agilent Technologies, Santa Clara, CA, USA).

### 3.2. Wine Samples

Ten different wines varieties (five white and five red) were blended to create a representative white and red matrix to be used for the optimization of the extraction method. For the accelerated aging, 7 different commercial Gewürztraminer wines of the 2019 vintage and 7 different commercial Teroldego wines of the 2019 vintage were acquired from different wineries in Trentino Alto Adige region. The basic enological analysis can be found as Supplementary in Table S6.

### 3.3. Wine Aging

The wine bottles were opened under a N_2_ hood and aliquoted in two technical replicates into 50-mL clear glass bottles, avoiding any headspace, and then, the bottles were enclosed in vacuum bags. Internally to each bottle was placed a Pst3 oxygen sensor (Nomacorc SA, Thimister–Clemont, Belgium) to measure the dissolved oxygen, which was also the total packaging oxygen (TPO), because the bottles were full. For the accelerated aging, the samples were stored at 50 °C in a laboratory heater. Each wine sample was analyzed immediately after 2.5 (first replicate) and 5 (second replicate) weeks of accelerated aging. Since the oxygen sensors were placed internally, the measurement was carried out using luminescence technology optical fibre outside the glass bottle by using the NomaSense system (Nomacorc SA, Thimister Clemont, Belgium).

### 3.4. Sample Preparation and Extraction

Sample preparation and extraction of the free aroma compounds was performed according to the modification of the method described in [9,44]. Solid-phase extraction was initially performed using 3 different cartridges, namely Bond Elut ENV (Agilent, Santa Clara, CA, USA), Isolute^®^ ENV+ (Biotage, Uppsala, Sweden), and LiChrolut^®^ EN (Merk, Darmstadt, Germany), filled with 200 mg stationary phase and pre-conditioned with 4 mL dichloromethane, followed by 4 mL of methanol and 4 mL of model wine. A total of 50 mL of wine mixed with 100 μL of internal standard (n-heptanol 250 mg/L) was loaded onto the cartridge, which was then washed with 3 mL of water. The cartridges were dried for 10 min and tested as reported above. The validated method uses the Isolute^®^ ENV+ cartridge that was pre-conditioned, loaded and dried in the same way, and eluted with 2 mL dichloromethane directly into the injection vials.

### 3.5. MS-MS Optimization

The list of compounds was put together in order to include the most important chemical classes (varietals, fermentative, and aging) for wine aroma. The optimization of the MS/MS method was performed for all compounds, diluted in ethanol solution, and injected in EI and operated in MRM mode. The optimizer software (embedded in MassHunter Workstation) was used in order to acquire two or three MS/MS transitions and after that to select the best collision energy for each transition. The results with all the settings parameters are reported in Table 1.

### 3.6. GC-MS/MS Analysis

The instrument method was adapted from [10] with some modification, using the Agilent Intuvo 9000 system for fast GC coupled with an Agilent 7010B triple quadrupole mass spectrometer (Agilent Technologies, Santa Clara, CA, USA) equipped with an electronic ionization source operating at 70 eV. The separation was achieved by injecting 1 μL in split mode (1:10) into a DB-Wax Ultra Inert column (30 m × 0.25-mm id × 0.25-μm film thickness, Agilent Technology, Santa Clara, CA, USA). The initial temperature of the GC oven was 40 °C for 2 min, ramped up by 10 °C/min to reach 55 °C, then by 20°/min until 165 °C, by 40 °C/min to 240 °C for 1.5 min, and finally by 50 °C/min to 250 °C and kept at this temperature for additional 4 min (16 total runtime). Helium was used as carrier gas (with a flow of 1.2 mL/min). The mass spectra were acquired in multiple reaction monitoring mode. Nitrogen was used as the collision gas, with a flow of 1.5 mL/min in addition with Helium at 4.0 mL/min as quench gas. The transfer line and source temperature were set at 250 °C and 230 °C, respectively. The data acquisition and subsequent analyses were done using the MassHunter Workstation software.

### 3.7. Method Validation

Validation of the extraction and GC/MS/MS method was performed in terms of limit of detection, limit of quantification, linearity range, and inter- and intraday precision (Supplementary Table S2).

The limit of quantification was taken as the lowest point of the calibration curve, and the limit of detection was set at 1/3 times the LOQ. Linearity was studied by injecting each compound at different ranges for a total of 20 concentration points. A calibration curve was established for each of the 64 compounds. The linear calibration parameters were obtained using the least squares regression method. The squared correlation coefficient (R2) was used to estimate linearity. The precision of the method was determined by calculating the coefficient of variation (CV) for daily (intraday) and day-to-day (interday) analysis using the medium spike level and the retention time. The recovery was tested using 3 different spike-level (low, medium, and high) standard solutions. Concentrations were referred to 2 mL in vial. The calculation was expressed by the following formula: Recovery% = [((spiked wine) − wine)/(solvent + spike)] × 100.

### 3.8. Statistical Analysis

The descriptive and ANOVA statistical analysis, and the visualization of the results was performed using SPSS V28 (IBM Statistics). The one-way ANOVA analysis was performed to compare the three groups’ means (t0, time zero; t1, 2.5 weeks; and t2, 5 weeks) for each measured compound. For the post hoc multiple comparison, the Tukey’s HSD statistical analysis were performed considering as a hypothesis with a *p*-value less than 0.05.

## 4. Conclusions

The study made it possible to identify the best cartridge to allow the main volatile compounds present in wine to be extracted repetitively and accurately. It then made it possible to reduce the volumes of solvents necessary for the preparation of the sample considerably and to elute directly into the vial for injection, avoiding any concentration step. The use of a triple quadrupole also made it possible to reduce the analysis time. Using this method, seven white and seven red wines were analyzed before and after accelerated aging. The analysis allowed us to monitor the behavior of the most important classes of volatile compounds that change during aging, finding many confirmations, such as the hydrolysis of non-volatile glycosidic precursors as well as the chemical rearrangements of certain terpene compounds with the formation of new impact molecules that are sometimes very important for aging red wine aroma; it will be necessary to test whether these notes are also appreciated in aromatic white wines. For other compounds, the analysis confirmed the already well-known behavior: the hydrolysis of acetates and linear ethyl esters, with consequent loss of fruity notes and the increase of some branched esters, which, especially in red wines, support the fruity note. New observations that will need to be explored also emerged, such as the high increase in safranal, a C10 norisoprenoid, during aging. The precursor of this compound in wine is not already known. From the results, it is also evident that many compounds reached the maximum quantity already after 2.5 weeks at 50 °C; however, studies at lower temperatures will be necessary to better understand these trends. The preliminary results obtained in the experiments of accelerated aging are promising and suggest that the method here employed could represent an affordable analytical tool in the quest to predict the aromatic potential during aging. We are aware that further work is needed, but a step has been made towards the validation of a protocol that could support winemakers in the selection of the wine lots suitable to produce reserve wine.

## Figures and Tables

**Table 1:** Compounds and analytical parameters (RT, retention time; R, compounds validated in red wine; W, compounds validated in white wines, CAS number, quantifier, and qualifier transition with collision energy (CE) used, ratio (qualifier/quantifier ± 20%), calibration curve, and linearity obtained for the studied compounds. (* linearity using split 1:150).

					Quantifier		Qualifier 1			Calibration Curve		Linearity (μg/L) Split 1:10 (* Split 1:150)	
Compound	RT	R	W	CAS Number	Q	CE V	q1	CE V	q1/Q	Equation	R^2^	LOQ	Max
Isobutyl acetate	4.527	×	×	110-19-0	56 > 41	9	56 > 39	21	0.32	y = 0.670244 * x + 4.653750 × 10^−6^	0.996162	0.5	750
Ethyl butyrate	4.768	×	×	105-54-4	71 > 43	5	116 > 73	11	0.04	y = 1.230283 * x + 2.590229 × 10^−4^	0.996698	0.1	600
Ethyl 2-methylbutyrate	4.945	×	×	7452-79-1	102 > 74	6	102 > 56	14	0.1	y = 0.862654 * x + 2.666487 × 10^−5^	0.993428	0.05	600
Ethyl isovalerate	5.110	×	×	108-64-5	88 > 61	4	85 > 57	4	1.46	y = 0.427345 * x + 1.411355 × 10^−4^	0.997103	0.15	250
Butyl acetate	5.151	×	×	123-86-4	56 > 41	8	56 > 39	21	0.32	y = 0.728005 * x + 2.214900 × 10^−4^	0.99692	0.5	600
Isopentyl acetate	5.678	×		123-92-2	70 > 55	7	55 > 29	9	0.21	y = 0.844247 * x + 2.784195 × 10^−4^	0.994801	0.5	600 *
Ethyl valerate	5.806	×	×	539-82-2	85 > 57	3	101 > 73	5	0.43	y = 0.346956 * x + 1.479302 × 10^−5^	0.995213	0.075	600
1,8-Cineole	6.552	×	×	470-82-6	154 > 84	8	154 > 69	21	0.78	y = 0.156753 * x + 2.082461 × 10^−5^	0.998566	0.05	380
Ethyl capronate	6.738	×	×	123-66-0	88 > 61	13	101 > 73	5	1.4	y = 0.301412 * x + 1.109648 × 10^−4^	0.993592	0.5	1500 *
Hexyl acetate	7.068	×	×	142-92-7	56 > 41	10	61 > 43	13	0.46	y = 0.766427 * x + 9.023786 × 10^−4^	0.998808	0.5	600
Ethyl heptanoate	7.561	×	×	106-30-9	113 > 43	8	113 > 57	5	0.2	y = 0.351393 * x + 5.145851 × 10^−5^	0.994433	0.1	250
*cis* Rose oxide	7.752	×	×	16409-43-1	139 > 69	12	154 > 112	4	0.01	y = 2.264015 * x − 1.932679 × 10^−5^	0.997857	0.055	364
*trans*-3-Hexen-1-ol	7.784	×	×	928-97-2	82 > 67	6	82 > 41	22	0.21	y = 0.886908 * x − 5.399564 × 10^−6^	0.99977	0.075	380
*trans* Rose oxide	7.879	×	×	16409-43-1	139 > 69	12	154 > 139	4	0.09	y = 2.327479 * x − 6.575159 × 10^−6^	0.997533	0.014	163
*cis*-3-Hexen-1-ol	7.969	×	×	928-96-1	82 > 67	6	82 > 41	22	0.21	y = 0.737803 * x + 1.343361 × 10^−4^	0.996857	0.1	500
Furfurylthiol	8.311	×	×	98-02-2	114 > 81	5	114 > 53	23	0.15	y = 0.914263 * x − 0.025057	0.995537	25	600
Ethyl caprylate	8.321	×	×	106-32-1	88 > 61	8	88 > 60	8	0.64	y = 0.535577 * x + 3.897772 × 10^−4^	0.996045	0.25	1500 *
Linalool oxide A	8.431	×	×	60047-17-8	94 > 79	9	111 > 93	3	0.48	y = 0.880633 * x + 1.053171 × 10^−5^	0.998926	0.136	326
1-Heptanol (ISTD)	8.450	×	×	111-70-6	70 > 55	8	70 > 42	4	0.32	-	-	-	-
Linalool oxide B	8.624	×	×	60047-17-8	94 > 79	10	111 > 93	3	0.43	y = 0.901968 * x − 2.790951 × 10^−5^	0.998762	0.114	274
2-sec-Butyl-3-methoxypyrazine	8.804	×	×	24168-70-5	138 > 123	11	151 > 83	9	0.13	y = 0.572936 * x − 2.320136 × 10^−5^	0.999409	0.05	380
Benzaldehyde	9.004	×	×	100-52-7	105 > 77	13	106 > 77	22	0.59	y = 2.520564 * x + 0.013265	0.997263	0.1	500
Ethyl leucate	9.021	×	×	10348-47-7	87 > 69	2	87 > 41	15	0.32	y = 0.738923 * x + 1.965922 × 10^−5^	0.999635	0.075	500
Linalool	9.035	×	×	126-91-0	93 > 77	14	93 > 91	14	0.57	y = 0.712852 * x + 1.982497 × 10^−5^	0.998334	0.15	500
Terpinen-4-ol	9.472	×	×	20126-76-5	93 > 77	14	136 > 93	8	0.2	y = 0.738923 * x + 1.965922 × 10^−5^	0.999635	0.075	380
Ethyl caprate	9.607	×	×	110-38-3	157 > 87	11	88 > 61	4	4	y = 0.110673 * x + 2.153586 × 10^−4^	0.997827	0.5	75 *
Benzylmercaptan	9.610	×	×	100-53-8	124 > 91	4	91 > 65	17	0.73	y = 1.697011 * x − 0.007896	0.990931	2.5	125
Phenylacetaldehyde	9.669		×	122-78-1	120 > 91	12	91 > 65	16	2.06	y = 0.640671 * x + 7.711668 × 10^−4^	0.999613	0.5	380
Safranal	9.742	×	×	116-26-7	150 > 121	5	91 > 65	15		y = 0.424750 * x − 1.216147 × 10^−5^	0.998124	0.1	500
Diethyl succinate	9.809	×	×	123-25-1	129 > 101	4	129 > 73	14	0.3	y = 1.799192 * x + 8.010092 × 10^−4^	0.999379	1	5000 *
α-Terpineol	9.954	×	×	7785-53-7	93 > 77	18	121 > 91	19	0.17	y = 0.883463 * x + 1.341620 × 10^−4^	0.997403	0.1	500
Valeric acid	10.099		×	109-52-4	60 > 42	11	73 > 55	9	0.45	y = 1.063399 * x − 0.006555	0.994681	5	120
β-Citronellol	10.223	×	×	7540-51-4	95 > 67	9	156 > 95	7	0.06	y = 0.234660 * x + 4.673063 × 10^−4^	0.997602	0.5	500
TDN	10.236	×	×	30364-38-6	157 > 142	14	172 > 157	9	0.42	y = 0.318269 * x + 1.753377 × 10^−4^	0.990574	1	125
Ethyl phenylacetate	10.371	×	×	101-97-3	164 > 91	6	164 > 105	3	0.15	y = 1.103282 * x + 1.327135 × 10^−5^	0.999731	0.05	380
Methyl salicylate	10.386	×	×	119-36-8	120 > 92	10	120 > 64	24	0.25	y = 2.769435 * x + 2.479293 × 10^−4^	0.996217	0.05	500
Nerol	10.393	×	×	106-25-2	136 > 121	5	121 > 105	9	1.45	y = 0.030762 * x + 1.555425 × 10^−5^	0.997223	1	500
Phenylethyl acetate	10.507	×	×	103-45-7	104 > 78	14	91 > 65	15	0.17	y = 2.617976 * x + 1.920198 × 10^−4^	0.999673	0.05	380
β-Damascone	10.539	×	×	23726-91-2	177 > 149	9	123 > 81	9	2.34	y = 0.189745 * x + 9.228603 × 10^−7^	0.999728	0.25	380
β-Damascenone	10.552	×	×	23726-93-4	190 > 121	5	190 > 175	6	0.7	y = 0.195451 * x − 3.627043 × 10^−6^	0.999509	0.1	380
Ethyl laurate	10.557	×		106-33-2	101 > 73	5	88 > 61	4	0.81	y = 1.069797 * x + 3.037786 × 10^−4^	0.997895	0.15	500
Geraniol	10.569	×	×	106-24-1	93 > 77	15	123 > 81	10	0.23	y = 0.112392 * x − 1.567887 × 10^−5^	0.995244	0.5	500
Guaiacol	10.675	×	×	90-05-1	109 > 81	10	109 > 53	21	0.18	y = 1.724700 * x + 7.658686 × 10^−4^	0.995602	0.15	500
Benzyl alcohol	10.732	×	×	100-51-6	108 > 79	16	108 > 77	32	0.36	y = 1.450902 * x + 0.001039	0.99974	0.1	380
*trans*-Whiskey lactone	10.799	×	×	39212-23-2	99 > 71	2	87 > 69	2	0.3	y = 1.236874 * x − 5.675139 × 10^−5^	0.99967	0.085	216
γ-Octalactone	10.938	×	×	104-50-7	85 > 57	5	100 > 72	3	0.05	y = 1.209842 * x − 1.267947 × 10^−4^	0.999779	0.25	380
β-Ionone	10.994	×	×	79-77-6	177 > 162	17	177 > 147	23	0.98	y = 0.643938 * x + 1.506262 × 10^−5^	0.999647	0.05	380
*cis*-Whiskey lactone	11.062	×	×	39212-23-2	99 > 71	2	87 > 69	2	0.5	y = 1.188140 * x − 6.218725 × 10^−5^	0.996834	0.108	216
Benzothiazole	11.110	×	×	95-16-9	135 > 91	17	108 > 82	20	0.66	y = 0.633120 * x + 3.161581 × 10^−4^	0.996517	0.25	500
4-Ethyl guaiacol	11.253	×	×	2785-89-9	137 > 94	21	152 > 137	12	0.7	y = 1.417789 * x − 2.338362 × 10^−5^	0.996422	0.15	500
Octanoic acid	11.314	×	× *	124-07-2	60 > 42	13	73 > 55	10	0.82	y = 0.818348 * x + 5.533180 × 10^−4^	0.997182	2.5	1500 *
γ-Nonalactone	11.328	×	×	104-61-0	128 > 95	6	128	0.04	0.99	y = 0.077137 * x − 5.774876 × 10^−6^	0.999203	0.5	380
Ethyl cinnamate	11.627	×	×	103-36-6	131 > 77	23	176 > 131	8	0.22	y = 1.777345 * x − 9.711529 × 10^−6^	0.999203	0.05	380
Nonanoic acid	11.670	×	×	112-05-0	60 > 42	12	129 > 87	6	0.39	y = 0.657491 * x − 0.001342	0.997586	5	380
4-Ethylphenol	11.706	×	×	123-07-9	122 > 107	11	107 > 77	16	1.76	y = 2.334519 * x + 4.080627 × 10^−5^	0.99959	0.05	380
Eugenol	11.723	×	×	97-53-0	164 > 149	9	164 > 104	13	0.55	y = 0.657689 * x − 2.787395 × 10^−4^	0.999095	0.5	380
γ-Decalactone	11.728	×	×	706-14-9	128 > 71	5	128 > 95	5	0.85	y = 0.133347 * x − 2.085701 × 10^−5^	0.999513	0.25	380
4-Vinylguaiacol	11.852	×	×	7786-61-0	135 > 107	5	150 > 135	22	0.76	y = 1.048017 * x − 0.002505	0.995304	2.5	600
δ-Decalactone	11.936	×	×	705-86-2	99 > 71	4	71 > 43	6	0.33	y = 1.394024 * x − 2.265714 × 10^−4^	0.990139	0.5	500
2-Aminoacetophenone	11.967	×	×	551-93-9	135 > 120	11	135 > 92	23	0.37	y = 1.785880 * x + 4.238632 × 10^−5^	0.99684	0.05	500
Decanoic acid	12.045	×	×	334-48-5	73 > 55	10	129 > 87	6	0.75	y = 0.561944 * x − 0.002159	0.998304	5	2500 *
Geranic acid	12.324	×	×	459-80-3	100 > 82	8	69 > 39	17	1.06	y = 0.140501 * x − 0.001569	0.997411	10	380
Menthalactone	12.448	×	×	13341-72-5	166 > 81	14	166 > 110	5	0.45	y = 0.555579 * x − 1.564872 × 10^−5^	0.997422	0.1	500 *
γ-Dodecalactone	12.624	×	×	2305-05-7	85 > 57	4	69 > 41	10	0.36	y = 0.930277 * x − 7.157235 × 10^−4^	0.997274	1	500
Zingerone	14.776	×	×	122-48-5	194 > 137	15	194 > 151	9	0.23	y = 0.854252 * x + 4.546328 × 10^−5^	0.99662	0.05	500

**Table 2:** Volatile compound concentrations (µg L^−1^) in Gewürztraminer wines during the experiment (t0 analyzed at time 0; t1 after 2.5 weeks; t2 after 5 weeks of accelerated aging at 50 °C. G, Gewürztraminer, 1–7 different wines; * compounds analyzed with split 1:150).

Time Point	t0							t1							t2						
Sample Code Compounds	G1	G2	G3	G4	G5	G6	G7	G1	G2	G3	G4	G5	G6	G7	G1	G2	G3	G4	G5	G6	G7
isobutyl acetate	49.70	43.09	60.40	53.73	36.66	31.96	47.51	24.80	39.33	33.14	39.85	23.61	27.09	28.00	18.51	34.82	20.65	35.64	19.11	21.34	23.87
ethyl butyrate	407.86	376.50	385.85	282.44	390.77	379.65	532.53	407.64	427.78	386.98	317.20	394.80	429.93	452.43	408.11	435.94	348.10	312.81	393.66	394.32	450.39
ethyl 2-methylbutyrate	16.79	7.07	5.86	16.38	8.34	6.03	7.90	33.90	17.56	12.66	35.09	18.71	14.81	15.82	43.84	24.17	13.54	39.93	24.37	16.82	20.70
ethyl isovalerate	35.87	14.18	9.80	31.92	16.59	11.64	14.08	72.26	37.94	23.48	72.40	38.51	30.89	30.49	92.76	52.22	25.67	81.72	49.95	35.56	40.10
butyl acetate	2.29	1.71	6.29	2.11	2.04	1.74	1.71	0.744	1.27	3.02	1.17	1.03	1.28	0.808	0.427	1.00	1.54	0.728	0.631	0.884	0.528
ethyl valerate	1.88	1.55	1.35	2.18	1.21	2.04	1.25	2.00	1.81	1.48	2.39	1.46	2.23	1.27	2.14	1.94	1.39	2.24	1.41	1.94	1.45
1,8-cineole	0.071	n.d.	n.d.	n.d.	n.d.	n.d.	0.066	3.65	1.14	1.32	2.68	2.14	1.02	3.47	6.44	2.36	1.84	3.88	3.67	1.80	5.00
ethyl capronate (*)	887.12	627.37	839.23	565.56	759.55	770.59	1017	641.15	582.64	631.28	449.45	641.25	662.52	773.39	683.74	558.69	638.59	366.36	611.46	506.69	733.75
hexyl acetate	121.64	50.58	152.05	51.17	48.99	29.10	47.29	34.10	28.12	51.32	17.22	21.14	14.08	18.13	15.99	18.05	30.29	6.96	9.68	7.46	10.19
ethyl heptanoate	0.943	1.41	1.02	1.34	0.970	1.84	0.907	0.574	1.06	0.610	0.906	0.800	1.28	0.687	0.659	1.02	0.579	0.666	0.689	0.955	0.680
*cis* rose oxide	3.77	4.41	2.84	2.91	3.57	5.24	6.69	2.58	3.89	2.06	2.15	2.89	4.45	5.61	2.53	3.46	1.72	1.64	2.55	3.41	4.78
*trans*-3-hexen-1-ol	145.83	47.11	74.97	54.99	79.77	53.95	57.23	123.30	41.20	70.10	49.37	70.73	46.47	68.84	120.10	39.03	64.80	46.70	67.11	44.62	64.78
*trans* rose oxide	0.380	0.667	0.382	0.312	0.448	0.922	1.08	0.251	0.366	0.218	0.218	0.285	0.416	0.505	0.246	0.331	0.184	0.175	0.251	0.323	0.427
*cis*-3-hexen-1-ol	51.65	15.36	68.75	20.46	26.57	25.26	29.01	43.72	13.49	62.42	18.30	24.17	21.71	33.97	43.23	13.26	60.82	17.64	23.05	20.83	32.24
ethyl caprylate (*)	1053	625.76	958.33	692.90	771.87	754.53	894.97	406.24	279.62	362.73	288.33	339.27	321.48	428.00	436.36	264.66	313.58	189.89	305.28	242.75	388.49
linalool oxide A	24.21	17.10	12.75	19.43	20.72	12.81	14.32	217.02	150.52	134.68	210.77	177.96	127.06	185.41	301.42	217.81	159.02	269.25	233.78	173.44	246.64
linalool oxide B	11.64	8.88	6.52	9.37	10.48	7.67	7.66	129.58	88.83	81.72	126.58	106.32	75.85	109.63	179.60	128.97	98.75	159.84	137.60	101.91	143.17
benzaldehyde	2.10	3.09	1.34	4.07	7.72	4.23	3.15	8.07	5.56	3.89	12.13	14.88	7.94	5.79	7.92	7.69	6.17	13.79	19.88	11.78	9.18
linalool	367.71	140.12	123.91	154.15	196.29	143.23	378.40	66.89	125.00	38.17	52.25	66.52	133.71	110.20	26.28	78.64	17.74	25.01	32.25	86.46	45.77
ethyl leucate	75.14	45.39	33.62	70.09	50.66	38.91	42.97	121.27	79.79	59.10	116.44	84.57	65.81	89.19	137.10	95.40	59.69	127.84	97.19	76.71	105.88
terpinen-4-ol	2.81	2.23	2.05	3.27	3.13	1.70	2.12	8.62	9.52	5.21	8.32	9.46	7.20	9.67	7.59	9.30	4.04	6.77	8.21	6.94	8.96
ethyl caprate	207.84	93.60	240.73	146.40	169.24	112.94	233.19	45.03	16.80	53.46	31.49	32.39	22.12	44.01	43.86	17.10	27.24	14.82	27.04	15.16	34.18
phenylacetaldehyde	25.76	14.96	12.34	24.88	20.06	21.90	17.47	23.50	12.76	16.89	17.86	16.23	16.89	14.90	41.03	15.00	51.91	30.86	23.16	38.91	25.80
safranal	0.161	0.128	0.141	0.144	0.145	0.161	0.127	1.08	0.732	0.828	1.05	0.896	0.836	0.800	1.46	1.04	0.949	1.15	1.16	1.14	1.04
diethyl succinate (*)	3083	3991	2078	3709	3672	6924	1753	8086	6315	4246	7754	6674	9038	5329	11127	8328	5929	9998	8686	11045	7955
α-terpineol	138.07	64.98	55.31	73.08	90.92	64.31	144.29	397.02	298.50	190.75	283.36	305.72	291.85	472.38	323.73	330.57	156.21	236.26	273.95	314.13	425.30
valeric acid	25.93	25.57	26.47	29.89	20.11	26.04	17.06	24.64	23.45	24.11	28.51	22.98	26.58	20.15	22.60	21.54	26.44	25.39	18.61	24.54	18.75
α-citronellol	43.44	97.69	30.66	45.07	63.06	107.05	98.73	10.61	43.45	10.73	9.66	18.97	51.22	33.81	6.03	26.47	8.87	5.59	10.72	31.95	18.68
TDN	1.37	0.696	0.808	1.11	0.990	0.589	0.553	16.88	7.35	17.78	17.20	15.97	8.13	9.05	21.15	11.08	13.30	11.02	17.60	9.53	9.75
ethyl phenylacetate	6.84	6.49	6.41	9.51	6.93	10.54	5.17	11.81	12.21	12.69	17.24	13.20	19.21	9.61	14.46	15.50	13.73	19.42	16.11	23.41	11.95
methyl salicylate	0.948	1.05	0.676	0.992	1.42	0.918	2.07	1.81	1.87	1.09	2.03	2.77	1.13	2.34	1.77	1.92	1.06	1.75	2.33	1.15	2.18
nerol	34.74	50.15	23.09	21.49	46.49	84.22	206.48	3.57	10.58	n.d	n.d	3.42	9.43	14.25	n.d	5.29	n.d	n.d	n.d	5.36	n.d
phenylethyl acetate	309.49	180.43	295.92	281.18	154.16	214.30	115.60	134.92	111.58	151.80	125.26	80.07	133.92	61.08	83.46	85.24	100.26	84.47	56.03	106.09	45.46
β-damascenone	2.11	1.86	2.57	3.35	1.78	2.43	2.65	4.31	2.20	3.93	4.46	2.75	2.87	2.76	4.64	2.27	3.89	4.36	3.06	3.45	3.24
geraniol	192.97	97.42	51.66	84.00	114.83	160.03	462.34	14.90	33.26	8.73	14.38	15.62	31.95	23.99	5.87	20.77	6.03	6.63	7.46	21.38	10.44
guaiacol	0.668	0.937	0.732	2.00	0.706	0.832	0.587	1.51	1.97	1.57	3.24	1.96	1.99	1.71	2.14	2.76	2.70	3.85	2.75	2.81	n.d
benzyl alcohol	190.26	147.24	91.30	78.59	100.32	173.85	109.19	159.93	122.57	82.55	71.09	90.25	149.30	121.54	163.21	123.39	106.34	70.65	89.08	148.97	117.21
*trans*-whiskey lactone	n.d	0.256	0.665	1.05	0.402	n.d	n.d	n.d	n.d	n.d	n.d	n.d	n.d	n.d	n.d	n.d	n.d	n.d	n.d	n.d	n.d
γ-octalactone	0.969	2.22	1.43	2.05	1.11	2.97	1.03	4.94	3.00	2.62	4.94	2.97	5.83	3.41	n.d	1.49	1.16	1.12	1.11	3.12	1.00
*cis*-whiskey lactone	n.d	0.322	1.03	1.72	0.719	0.478	n.d	n.d	n.d	1.02	1.23	n.d	n.d	n.d	n.d	0.634	0.831	1.86	0.678	0.618	8.39
benzothiazole	0.305	0.114	0.439	0.090	0.141	0.424	n.d	0.841	0.683	0.873	0.567	0.773	0.795	0.506	0.855	0.707	1.68	0.709	0.669	0.874	0.490
4-ethyl guaiacol	0.217	0.195	0.149	0.541	0.384	0.253	0.251	0.289	0.206	0.227	0.584	0.317	0.232	0.239	0.397	0.276	0.373	0.673	0.381	0.319	0.279
γ-nonalactone	4.60	11.05	7.56	7.74	6.54	17.60	6.46	4.57	10.34	7.37	7.85	6.50	16.35	5.03	4.88	11.81	13.19	8.72	7.00	17.96	5.91
ethyl cinnamate	0.990	0.783	1.39	0.841	0.706	0.966	0.596	0.765	0.485	0.979	0.683	0.714	0.762	0.372	0.791	0.619	0.945	0.613	0.570	0.789	0.442
nonanoic acid	71.10	44.38	36.35	58.65	54.77	47.11	33.04	108.32	83.46	68.51	100.68	96.69	81.26	75.45	107.48	89.85	87.52	98.33	101.69	86.67	80.57
γ-decalactone	1.29	1.73	1.45	1.57	0.966	2.35	2.04	1.21	1.86	1.58	1.55	1.13	2.25	1.94	1.28	1.75	1.52	1.52	1.09	2.09	1.90
4-ethyl-phenol	0.200	0.290	0.525	0.874	0.381	0.246	0.249	0.649	0.604	0.653	1.15	0.658	0.471	0.312	0.808	0.441	0.895	1.23	0.623	0.406	0.383
eugenol	5.55	4.13	5.26	3.66	4.89	5.48	7.98	6.88	4.63	6.19	4.78	5.68	6.15	8.20	7.01	4.86	5.73	4.76	5.83	6.17	8.47
4-vinylguaiacol	876.34	652.95	313.96	865.53	653.64	992.86	546.38	280.06	387.24	330.91	271.41	247.19	384.19	405.91	265.65	433.23	312.17	228.34	240.37	303.36	372.68
δ-decalactone	9.88	6.72	6.06	6.61	8.62	10.48	7.30	23.03	15.33	13.41	16.70	20.36	21.18	20.58	22.53	14.47	11.62	15.22	20.69	20.26	19.72
2-aminoacetophenone	0.173	0.268	0.270	0.192	0.225	0.271	0.222	0.324	0.500	0.350	0.453	0.663	0.306	0.416	0.340	0.805	0.433	0.483	0.927	0.406	0.934
decanoic acid (*)	2365	1201	3015	1363	1870	1280	2759	2485	1390	3635	1420	2138	1403	3016	2237	1297	3076	1218	1937	1339	2650
geranic acid (*)	333.07	361.97	208.37	228.28	336.24	377.52	623.64	472.68	488.59	292.25	397.00	427.23	521.25	561.39	389.52	453.40	230.24	346.42	373.55	507.17	479.70
γ-dodecalactone	46.45	39.74	19.52	19.94	57.89	135.39	44.56	29.38	23.14	14.16	15.82	39.66	111.84	29.49	32.47	25.06	49.70	13.17	35.73	81.24	24.15
zingerone	23.12	33.59	17.51	18.07	24.06	31.76	22.51	27.50	36.77	19.60	22.02	26.92	34.25	28.89	28.58	37.56	18.03	21.26	26.87	32.78	29.48

**Table 3:** Volatile compound concentrations (µg L^−1^) in Teroldego wines during the experiment (t0 analyzed at time 0; t1 after 2.5 weeks; t2 after 5 weeks of accelerated aging at 50 °C. T, Teroldego, 1–7 different wines; * compounds analyzed with split 1:150).

Time Point	t0							t1							t2						
Sample Code Compounds	T1	T2	T3	T4	T5	T6	T7	T1	T2	T3	T4	T5	T6	T7	T1	T2	T3	T4	T5	T6	T7
isobutyl acetate	57.41	59.48	55.33	58.84	51.22	58.79	58.57	46.21	53.39	50.93	56.65	64.27	64.95	58.69	48.81	52.45	50.68	58.28	71.77	64.35	60.14
ethyl butyrate	168.24	257.81	155.78	161.86	199.52	141.15	191.17	148.64	267.62	163.87	160.39	223.13	171.94	202.27	162.90	275.77	170.41	165.86	235.84	176.21	210.07
ethyl 2-methylbutyrate	10.57	6.15	23.96	12.11	12.81	15.78	12.51	16.10	11.52	37.60	19.91	23.60	31.60	21.10	21.75	14.77	47.16	26.57	31.53	41.16	26.99
ethyl isovalerate	20.69	12.82	33.38	21.43	18.53	27.15	17.66	31.03	25.58	51.80	36.44	36.22	56.22	30.64	41.49	32.42	64.85	48.39	48.34	73.11	39.59
butyl acetate	0.812	1.87	1.17	1.34	1.53	1.38	1.89	0.446	1.37	1.03	1.13	1.88	1.43	1.60	0.399	1.34	1.11	1.11	2.08	1.41	1.59
isopentyl acetate (*)	1134	1222	1045	851.92	534.52	966.93	764.53	583.50	754.77	619.80	559.72	477.84	716.72	529.70	545.52	630.17	511.77	475.04	477.22	585.28	460.93
ethyl valerate	0.779	2.05	0.939	0.872	1.36	0.662	3.19	0.625	1.97	0.957	0.869	1.37	0.765	3.11	0.738	2.07	1.06	0.961	1.42	0.862	3.27
1,8-cineole	n.d	n.d	n.d	n.d	n.d	n.d	n.d	n.d	0.067	0.079	n.d	n.d	n.d	0.064	0.087	0.104	0.119	0.071	0.068	0.080	0.102
ethyl capronate	308.90	341.96	228.02	260.40	199.22	228.13	245.68	186.99	243.66	168.32	184.84	155.52	213.41	191.81	210.96	230.38	173.81	193.49	154.97	217.36	190.41
hexyl acetate	18.18	15.93	14.99	9.24	1.90	7.12	5.87	10.71	12.54	7.90	4.51	1.97	5.58	5.49	6.34	5.90	5.09	3.43	1.67	3.42	3.03
ethyl heptanoate	0.847	0.949	0.702	0.539	1.04	0.407	1.15	0.400	0.523	0.411	0.299	0.604	0.323	0.699	0.449	0.448	0.438	0.311	0.600	0.348	0.661
*trans*-3-hexen-1-ol	51.82	36.95	23.29	46.12	25.49	49.66	21.53	43.11	32.78	21.42	41.74	23.86	48.54	20.48	43.33	31.92	20.94	41.87	23.00	44.73	20.12
*cis*-3-hexen-1-ol	217.23	189.21	102.48	160.01	92.86	173.96	83.17	175.85	167.27	93.11	142.44	86.85	167.20	77.59	177.19	163.65	91.52	143.41	85.29	157.79	77.62
ethyl caprylate	323.11	349.92	311.94	269.26	184.95	198.17	234.68	97.42	101.24	106.61	79.33	65.28	91.43	87.01	95.62	84.14	104.01	83.78	60.55	90.15	78.39
linalool oxide A	3.14	1.72	3.62	2.49	3.99	10.71	3.59	11.27	7.45	9.58	8.91	11.59	21.67	11.66	13.91	9.21	12.40	12.19	14.78	24.80	15.23
linalool oxide B	1.61	1.09	1.81	1.37	2.35	5.94	2.14	6.39	4.53	5.25	5.15	6.84	12.41	6.91	7.93	5.54	6.83	7.06	8.65	14.15	8.88
benzaldehyde	3.72	13.08	25.88	5.05	17.20	2.93	12.71	4.28	13.80	36.00	4.89	16.72	2.75	17.15	5.94	12.89	36.19	4.95	15.82	3.46	14.91
linalool	12.19	13.96	12.68	9.72	12.27	10.54	11.82	4.22	5.62	3.31	3.79	7.18	3.35	5.17	3.02	3.71	2.05	2.26	4.34	1.97	3.21
ethyl leucate	157.61	70.26	92.70	86.28	86.70	101.36	103.64	174.37	82.24	119.71	114.53	121.85	138.63	131.78	183.80	83.39	127.10	121.97	134.37	144.32	140.05
terpinen-4-ol	0.547	1.96	0.280	0.426	0.413	0.352	0.678	0.589	1.62	0.363	0.386	0.482	0.341	0.688	0.582	1.31	0.353	0.388	0.473	0.338	0.598
ethyl caprate	65.81	64.87	117.89	67.48	30.91	25.45	27.53	6.42	5.10	14.91	6.52	3.72	3.92	3.84	6.24	3.56	10.53	6.84	3.36	3.95	2.42
safranal	0.141	0.148	0.150	0.137	0.121	0.111	0.143	0.807	0.781	0.737	0.640	0.825	0.745	0.865	1.04	0.926	0.973	0.862	1.11	0.928	1.12
α-terpineol	4.63	5.32	7.27	4.06	3.65	5.10	4.05	12.45	14.52	12.44	10.49	12.84	11.24	12.25	12.49	14.24	11.95	10.42	14.42	10.61	12.76
β-citronellol	21.84	23.93	12.16	14.26	18.56	13.17	12.92	8.93	12.97	6.36	8.88	11.06	6.62	6.38	6.64	6.43	2.65	3.81	7.39	2.80	4.95
TDN	0.399	0.267	0.704	0.262	n.d	n.d	0.477	5.25	2.73	4.93	2.72	3.40	3.73	7.85	5.75	3.58	5.95	4.62	5.86	6.20	8.91
ethyl phenylacetate	9.47	5.61	12.23	6.48	9.36	9.21	12.33	13.24	8.54	17.11	9.85	13.91	13.71	18.48	15.82	9.85	20.01	12.01	16.93	16.24	22.41
methyl salicylate	2.73	1.72	1.99	2.24	6.25	4.70	3.13	2.74	1.72	2.27	2.68	6.38	4.88	3.56	2.72	1.67	2.26	2.71	6.41	4.82	3.43
nerol	7.63	3.99	2.59	2.43	9.77	4.00	n.d	n.d	n.d	n.d	n.d	n.d	n.d	n.d	n.d	n.d	n.d	n.d	n.d	n.d	n.d
phenylethyl acetate	162.60	97.59	205.32	74.60	74.12	134.05	90.11	98.89	65.08	136.79	56.08	74.53	94.64	67.30	87.60	55.74	113.51	50.94	79.06	81.61	61.16
β-damascenone	2.61	2.84	2.90	2.00	2.55	2.00	1.95	2.98	3.94	4.57	2.98	4.32	2.88	2.95	3.59	3.76	4.44	3.04	4.53	3.68	2.67
ethyl laurate	2.25	1.55	1.85	1.42	1.26	0.634	0.597	n.d	n.d	n.d	n.d	n.d	n.d	n.d	n.d	n.d	n.d	n.d	n.d	n.d	n.d
geraniol	6.59	7.00	3.63	4.71	10.99	5.85	9.38	1.57	2.00	1.35	1.50	3.11	1.23	1.97	1.19	1.40	n.d	1.06	1.72	n.d	1.31
guaiacol	2.49	4.44	4.79	3.54	6.78	5.14	9.28	12.78	20.61	16.16	18.42	28.24	18.89	23.28	16.00	25.04	21.09	25.02	37.36	23.93	28.78
benzyl alcohol	158.67	145.93	453.65	148.69	166.99	283.95	174.16	139.25	139.53	422.92	143.42	165.20	275.95	169.38	149.14	140.61	435.27	151.65	175.04	283.55	178.19
*trans*-whiskey lactone	3.99	0.453	0.397	1.22	8.39	7.80	23.91	3.69	0.345	0.387	1.27	8.44	7.82	23.56	3.75	0.349	0.406	1.28	8.88	8.18	24.25
γ-octalactone	0.694	0.801	0.613	0.546	0.853	0.778	1.04	1.02	0.885	0.802	0.652	1.43	1.12	1.08	0.929	0.709	0.918	0.724	1.25	0.983	1.23
β-ionone	0.123	0.228	0.101	0.112	0.100	0.133	0.088	0.097	0.139	0.088	0.104	0.096	0.119	0.051	0.096	0.101	0.073	0.085	0.090	0.113	n.d
*cis*-whiskey lactone	3.17	0.910	1.08	2.81	21.37	12.83	57.68	2.96	0.704	0.999	3.07	21.63	12.77	56.68	3.13	0.645	0.945	3.27	22.13	12.72	58.45
benzothiazole	0.231	0.094	0.739	n.d	1.23	n.d	1.03	0.650	0.609	1.21	0.550	1.82	0.501	1.42	0.690	0.460	1.12	0.463	1.78	0.494	1.38
4-ethyl guaiacol	0.562	2.21	0.438	0.970	0.662	1.58	10.52	0.594	2.20	0.495	1.07	0.751	1.64	10.99	0.598	2.24	0.536	1.10	0.782	1.67	11.26
γ-nonalactone	6.27	9.04	5.56	4.79	13.65	6.75	13.34	6.60	9.68	6.57	5.97	15.71	7.78	14.93	7.01	10.76	7.14	6.41	16.91	8.16	15.96
octanoic acid (*)	3103	2737	2333	2330	1623	2030	2027	3244	3268	2779	2751	1988	2384	2467	3110	2976	2420	2458	1808	2173	2183
ethyl cinnamate	0.543	1.11	1.03	0.444	1.91	0.647	0.921	0.383	0.994	0.766	0.345	1.56	0.514	0.488	0.440	0.813	0.858	0.333	1.57	0.511	0.871
nonanoic acid	72.39	73.51	107.82	87.43	69.62	102.95	84.94	94.79	80.77	118.66	113.05	90.22	120.94	98.51	90.90	83.22	115.94	108.99	93.67	114.98	101.40
γ-decalactone	0.951	1.00	0.859	0.277	0.979	0.494	0.962	0.89	0.79	0.72	0.40	1.04	0.490	0.831	0.926	0.747	0.683	0.383	1.05	0.436	0.885
4-ethyl-phenol	8.01	12.79	1.96	7.71	1.41	26.11	192.31	8.34	13.56	2.08	8.64	1.64	28.37	207.30	8.38	12.84	1.99	8.38	1.63	27.28	199.22
eugenol	3.67	4.18	2.75	2.92	8.62	7.21	10.32	3.95	4.75	3.38	3.40	9.48	7.72	11.31	4.19	4.83	3.47	3.46	9.62	7.69	11.59
4-vinylguaiacol	6.69	7.89	7.78	5.23	5.17	7.05	11.32	14.36	16.38	11.73	9.55	11.10	11.39	15.89	17.13	20.12	16.54	13.34	16.43	15.97	20.55
δ-decalactone	5.00	7.19	7.07	5.80	7.84	6.92	7.72	6.04	6.97	8.74	7.35	10.16	7.52	11.71	7.43	7.38	9.20	7.87	11.47	10.97	12.56
2-aminoacetophenone	0.226	0.408	0.243	0.257	0.228	0.254	0.266	0.208	0.236	0.210	0.220	0.216	0.198	0.235	0.164	0.208	0.198	0.175	0.155	0.172	0.236
decanoic acid (*)	946.90	629.04	1137	944.74	444.79	438.11	371.66	841.86	643.68	1227	899.36	461.66	440.14	396.50	820.80	610.97	1078	829.52	431.54	417.26	347.55
geranic acid	37.38	38.16	30.08	33.64	39.38	37.50	28.70	39.57	45.71	33.96	38.64	44.03	39.73	34.48	36.08	38.95	28.63	30.66	36.26	32.12	32.22
menthalactone	n.d	n.d	n.d	n.d	n.d	n.d	n.d	n.d	n.d	n.d	n.d	n.d	n.d	n.d	n.d	n.d	n.d	n.d	n.d	n.d	n.d
γ-dodecalactone	37.83	28.49	27.52	21.13	35.04	21.57	34.44	19.68	18.00	16.78	12.96	23.70	11.42	24.70	24.90	18.56	16.22	14.45	25.63	12.05	25.86
zingerone	0.505	2.26	0.690	0.579	0.334	1.58	1.26	0.693	0.541	0.547	0.767	0.491	1.84	0.979	0.771	0.597	0.532	0.921	0.525	1.82	1.03

## Data Availability

All data is contained within the article and Supplementary Materials.

## Supplementary Materials

The following supporting information can be downloaded at: https://www.mdpi.com/article/10.3390/metabo12020180/s1, Figure S1: Elution tests from cartridges MI (1st extraction) and MII (2nd extraction) with 3 aliquots of DCM solvent (1, 2, 3) Cartridge B = Bond Elut ENV; I = Isolute® ENV+; L = LiChrolut® EN; W = white wine; M = medium spike, Table S1: Comparison of cartridges; percentage of compounds found in the 2nd and 3rd solvent fractions (dichloromethane) considering 100% the content of the 1st fraction. Descriptive statistics, one-way Anova analysis and post-hoc test (Tukey test *p* < 0.05) (*n* = 7 wine sample; 4 white and 3 red), Table S2: Recovery, intraday and interday precision for red and white wines, in red compounds with unacceptable values, Table S3: Repeatability of the accelerated aging treatment of two different Gewürztraminer wines (A, B) kept for 4 days at T equal to 40 °C and then analyzed following the validated SPE-GC-MS/MS method, Table S4: Descriptive statistics of the measured volatile compounds and one-way Anova analysis and post-hoc test results for the Gewürztraminer wines, Table S5: Descriptive statistics of the measured volatile compounds and one-way Anova analysis results for the Teroldego wines, Table S6: basic enological analysis of commercial wines used for accelerating aging.
