# Supplementary material for: Measurement of the Effect of Accelerated Aging on the Aromatic Compounds of Gewürztraminer and Teroldego Wines, Using a SPE-GC-MS/MS Protocol
Source: Metabolites. 2022 Feb 15;12(2):180. doi: 10.3390/metabo12020180 (PMC8876733; doi:10.3390/metabo12020180)
Supplement: Supplementary file 1 [file metabolites-12-00180-s001.zip › metabolites-1540950-supplementary.pdf]

Measurement of the effect of accelerated aging on the aromatic compounds of Gewürztraminer and Teroldego wines, using a SPE-GC-MS /MS protocol

Silvia Carlin[1*], Cesare Lotti[1], Ludovica Correggi[2], Fulvio Mattivi[1,3], Panagiotis Arapitsas[1,4] and Urska Vrhovsek[1]

[1] Department of Food Quality and Nutrition, Edmund Mach Foundation, Research and Innovation Centre, Via Edmund Mach 1, 38010 San Michele all'Adige, TN, Italy;

[2] Department of Food and Drug, University of Parma, Area Parco delle Scienze 27/A, 43124, Parma, Italy

[3] Department of Cellular Computational and Integrative Biology, University of Trento, Via Sommarive 9, I - 38123 Povo, Trento, Italy

[4] Department of Wine, Vine and Beverage Sciences, School of Food Science, University of West Attica, Ag. Spyr-idonos str, Egaleo, 12243 Athens, Greece

* Correspondence: silvia.carlin@fmach.it; Tel.: +39-0461-615106

Supplementary Figure and Tables

Figure S1. Elution tests from cartridges MI (1st extraction) and MII (2nd extraction) with 3 aliquots of DCM solvent (1,2,3) Cartridge B= Bond Elut ENV; I= Isolute® ENV+; L= LiChrolut® EN; W= white wine; M=medium spike

Table S1. Comparison of cartridges; percentage of compounds found in the 2nd and 3rd solvent fractions (dichloromethane) considering 100% the content of the 1st fraction. Descriptive statistics, one-way Anova analysis and post-hoc test (Tukey test $p<0.05$) (N= 7 wine sample; 4 white and 3 red)

Table S2: Recovery, intraday and interday precision for red and white wines, in red compounds with unacceptable values.

Table S3. Repeatability of the accelerated aging treatment of two different Gewürztraminer wines (A, B) kept for 4 days at T equal to 40 °C and then analyzed following the validated SPE-GC-MS/MS method.

Table S4. Descriptive statistics of the measured volatile compounds and one-way Anova analysis and post-hoc test results for the Gewürztraminer wines.

Table S5. Descriptive statistics of the measured volatile compounds and one-way Anova analysis results for the Teroldego wines.

Table S6: basic enological analysis of commercial wines used for accelerating aging

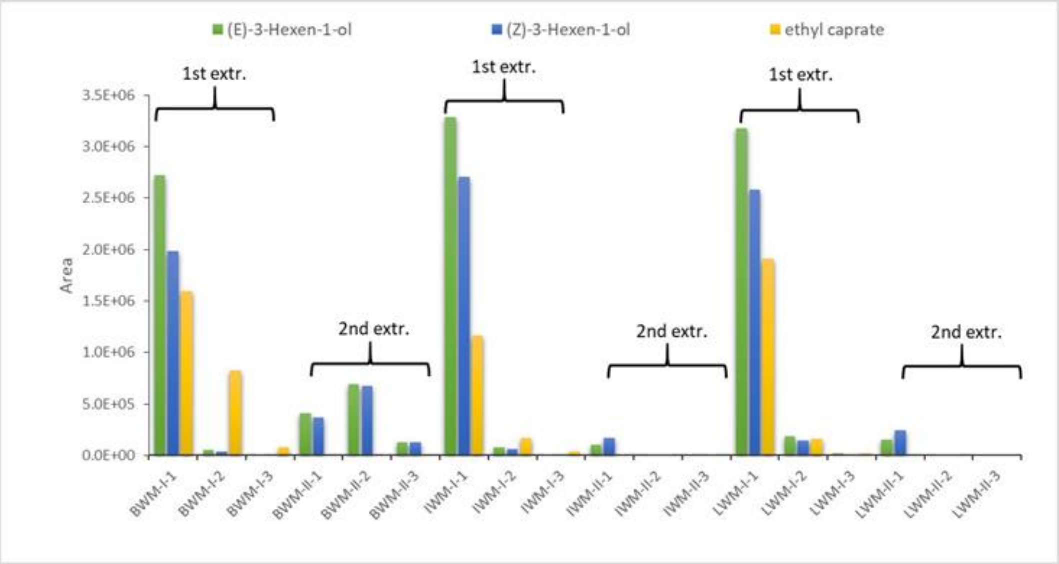

Figure S1. Elution tests from cartridges M_I (1st extraction) and M_{II} (2nd extraction) with 3 aliquots of DCM solvent (1,2,3) Cartridge B= Bond Elut ENV; I= Isolute® ENV+; L= LiChrolut® EN; W= white wine; M=medium spike

Table S1. comparison of cartridges; percentage of compounds found in the 2nd and 3rd solvent fractions (dichloromethane) considering 100% the content of the 1st fraction. Descriptive statistics and one-way Anova analysis and post-hoc test (Tukey test p<0.05) (N= 7 wine sample; 4 white and 3 red)

**Compounds**			**Bond Elut ENV**				**Isolute® ENV+**				**LiChrolut® EN**			
	**F**	**Significance**	**N**	**Mean**	**Std. Dev.**	**Tukey**	**N**	**Mean**	**Std. Dev.**	**Tukey**	**N**	**Mean**	**Std. Dev.**	**Tukey**
butyl acetate	3.81	0.041	7	4.83	3.78	**a**	7	2.40	3.09	**a,b**	7	0.55	1.23	**b**
isobutanol	7.18	<0.01	7	29.69	25.38	**a**	7	2.37	1.15	**b**	7	5.08	4.22	**b**
1-hexanol	4.03	0.035	7	17.39	2.87	**a**	7	12.29	8.05	**a,b**	7	8.74	5.05	**b**
trans-3-Hexen-1-ol	9.10	<0.01	7	13.23	7.88	**a**	7	3.75	1.97	**b**	7	3.28	2.61	**b**
cis-3-Hexen-1-ol	9.08	<0.01	7	13.66	8.64	**a**	7	3.38	1.39	**b**	7	3.13	2.63	**b**
methionol	9.09	<0.01	7	15.53	10.16	**a**	7	2.51	1.11	**b**	7	4.43	3.11	**b**
benzyl alcohol	6.18	<0.01	7	15.38	9.77	**a**	7	5.24	1.95	**b**	7	5.31	4.03	**b**
ethyl caprate	10.40	<0.01	7	78.87	27.69	**a**	7	31.81	32.33	**b**	7	17.44	16.56	**b**

Table S2. recovery, intraday and interday precision for red and white wines, in red compounds with unacceptable values.

	RED WINE			WHITE WINE		
	Recovery % (n=10)	CV % INTRADAY (n=5)	CV% INTERDAY (n= 5)	Recovery % (n=10)	CV % INTRADAY (n=5)	CV% INTERDAY (n= 5)
Isobutyl acetate	87.5	6.1	1.9	88.7	1.1	3.7
Ethyl butyrate	86.5	5.7	2.0	81.3	0.6	3.1
Ethyl 2-methylbutyrate	93.1	7.2	4.8	91.2	2.6	3.7
Ethyl isovalerate	87.9	6.3	2.2	88.1	1.5	4.0
Butyl acetate	90.8	6.3	2.2	92.5	1.5	3.2
Isobutanol	-26.8	5.9	7.2	-31.2	2.3	9.9
Isopentyl acetate	83.4	5.2	2.3	56.7	0.7	2.4
Ethyl valerate	90.4	6.0	2.2	91.9	1.4	4.0
1,8-Cineole	96.1	6.2	1.7	93.2	1.7	2.0
Ethyl capronate	83.7	5.2	1.7	63.7	0.9	2.9
Hexyl acetate	90.4	5.4	2.7	86.9	1.1	2.8
Acetoin	-1.2	24.8	24.2	1.1	3.3	7.2
Ethyl heptanoate	85.6	5.9	1.9	83.0	1.5	4.5
Ethyl lactate	-384.8	4.9	2.1	-75.6	2.0	2.7
1-Hexanol	58.5	4.8	1.9	52.9	0.4	1.9
cis Rose oxide	96.6	5.8	2.2	93.0	1.3	2.3
trans-3-Hexen-1-ol	79.5	4.9	5.3	95.8	1.4	2.9
trans Rose oxide	97.2	6.0	2.4	92.9	1.4	2.6
cis-3-Hexen-1-ol	71.2	4.6	6.2	90.5	1.6	2.7
Ethyl caprylate	78.7	5.3	3.2	67.7*	1.2	4.4
Furfurylthiol	59.5*	9.3	16.0	71.4	2.4	10.5
Linalool oxide A	97.7	6.0	2.4	99.4	1.0	1.0
Linalool oxide B	96.9	6.2	2.6	98.8	1.2	1.3
2-sec-Butyl-3-methoxypyrazine	95.5	6.1	2.2	92.2	1.6	2.0
Benzaldehyde	76.1	6.0	3.3	60.3*	4.9	7.8
Linalool	98.4	5.9	2.3	94.5	0.6	1.0
Ethyl leucate	94.9	5.2	1.6	97.9	1.0	1.2
Isobutyric acid	6.2	5.3	4.1	6.6	2.0	2.2
Terpinen-4-ol	96.0	6.3	2.2	94.5	1.4	1.8
Butyric acid	6.5	5.4	4.7	0.8	1.5	2.3
Ethyl caprate	63.6	7.6	5.1	62.4*	2.1	5.0
Benzylmercaptan	68.8	7.7	9.8	79.8	2.2	7.9
Phenylacetaldehyde	27.8	16.3	39.6	54.2	5.1	15.2
Safranal	94.8	5.9	2.5	91.6	1.4	2.8
Isovaleric acid	9.0	4.7	4.2	29.1	1.8	2.9
Diethyl succinate	-17.8	4.9	2.6	46.2	0.8	1.8
α-Terpineol	97.9	6.3	2.6	93.8	1.5	1.7
Methionol	-3.3	5.4	5.3	10.4	2.7	7.3
Valeric acid	50.9	5.1	11.1	78.2	2.7	3.1

	RED WINE			WHITE WINE		
	**Recovery % (n=10)**	**CV % INTRADAY (n=5)**	**CV% INTERDAY (n= 5)**	**Recovery % (n=10)**	**CV % INTRADAY (n=5)**	**CV% INTERDAY (n= 5)**
**β-Citronellol**	**95.5**	**5.6**	**2.7**	**92.8**	**1.4**	**2.4**
**TDN**	**69.9**	**7.1**	**3.5**	**62.6**	**2.8**	**6.7**
**Ethyl phenylacetate**	**96.9**	**6.3**	**2.6**	**95.3**	**0.8**	**1.5**
**Methyl salicylate**	**95.7**	**6.3**	**2.5**	**95.0**	**1.4**	**1.5**
**Nerol**	**94.9**	**6.0**	**3.7**	**91.3**	**1.7**	**2.8**
**Phenylethyl acetate**	**92.9**	**5.9**	**2.4**	**82.4**	**0.6**	**1.5**
**β-Damascone**	**90.3**	**6.3**	**3.2**	**78.8**	**1.4**	**1.7**
**Hexanoic acid**	**-2.1**	**5.4**	**2.5**	**-48.3**	**0.7**	**1.7**
**β-Damascenone**	**87.7**	**6.0**	**3.0**	**74.3**	**1.4**	**3.6**
**Ethyl laurate**	**62.7***	**9.5**	**8.0**	**44.4**	**5.4**	**15.7**
**Geraniol**	**91.9**	**5.8**	**3.3**	**87.6**	**1.1**	**2.0**
**Guaiacol**	**85.9**	**6.3**	**3.4**	**98.4**	**1.5**	**3.0**
**Benzyl alcohol**	**60.9***	**5.0**	**3.8**	**66.7**	**1.8**	**3.4**
***trans*-whiskey lactone**	**96.6**	**5.9**	**3.4**	**93.0**	**1.5**	**3.0**
**2-Phenylethanol**	**-135.6**	**5.7**	**4.6**	**6.2**	**1.0**	**2.1**
**γ-octalactone**	**96.6**	**6.4**	**3.2**	**96.2**	**1.4**	**1.6**
**β-ionone**	**92.7**	**5.4**	**3.8**	**88.0**	**1.7**	**1.8**
***cis*-whiskey lactone**	**95.5**	**5.5**	**2.7**	**95.3**	**1.7**	**2.4**
**Benzothiazole**	**93.5**	**6.6**	**2.0**	**94.9**	**2.8**	**2.7**
**4-Ethyl guaiacol**	**95.6**	**6.5**	**3.2**	**90.7**	**1.4**	**1.3**
**Octanoic acid**	**60.3***	**5.6**	**2.0**	**72.3***	**0.9**	**1.6**
**γ-Nonalactone**	**93.8**	**5.6**	**3.2**	**89.9**	**1.8**	**1.8**
**Ethyl cinnamate**	**93.2**	**5.5**	**4.6**	**89.6**	**1.6**	**1.3**
**Nonanoic acid**	**89.7**	**5.0**	**5.8**	**89.4**	**3.4**	**2.9**
**γ-decalactone**	**91.4**	**5.2**	**3.8**	**89.2**	**2.0**	**2.5**
**4-Ethyl-phenol**	**86.7**	**6.2**	**2.4**	**92.3**	**3.5**	**3.4**
**Eugenol**	**94.5**	**6.0**	**3.9**	**92.6**	**1.5**	**1.1**
**4-Vinylguaiacol**	**82.6**	**6.3**	**4.0**	**82.8**	**1.1**	**1.9**
**δ-decalactone**	**61.0**	**5.0**	**5.4**	**59.6**	**2.0**	**2.9**
**2-Aminoacetophenone**	**88.8**	**6.3**	**3.6**	**89.3**	**2.3**	**2.0**
**Decanoic acid**	**94.7**	**3.2**	**3.4**	**72.3***	**2.2**	**3.4**
**Geranic acid**	**89.0**	**5.0**	**3.7**	**81.3**	**2.8**	**4.3**
**Menthalactone**	**92.9***	**5.7**	**4.3**	**73.5**	**2.0**	**1.5**
**γ-dodecalactone**	**82.7**	**5.3**	**4.0**	**82.3**	**2.4**	**2.6**
**Zingerone**	**83.1**	**3.7**	**4.8**	**87.1**	**3.0**	**4.2**

*Compounds where recovery is improved by increasing the split (1:150)

Table S3. repeatability of the accelerated aging treatment of two different Gewürztraminer wines (A, B) kept for 4 days at T equal to 40 °C and then analyzed following the validated SPE-GC-MS/MS method.

	**Gewürztraminer A (40°C 4 days)**								**Gewürztraminer B (40°C 4 days)**							
	**GW A1**	**GW A2**	**GW A3**	**GW A4**	**GW A5**				**GW B1**	**GW B2**	**GW B3**	**GW B4**	**GW B5**			
**Compounds**						***mean***	***Std dev.***	***cv%***						***mean***	***Std dev.***	***cv%***
isobutyl acetate	58.3	56.2	55.5	55.5	56.8	*56.5*	*1.2*	***2.1***	46.9	46.5	46.7	46.7	46.6	46.7	0.2	**0.3**
ethyl butyrate	551	516	514	510	522	*523*	*16.4*	***3.1***	459	461	455	459	458	458	2.3	**0.5**
ethyl 2-methylbutyrate	3.7	3.4	3.4	3.4	3.5	*3.5*	*0.1*	***3.4***	3.4	3.4	3.3	3.3	3.3	3.3	0.0	**0.9**
ethyl isovalerate	6.4	6.0	6.0	5.9	6.1	*6.1*	*0.2*	***3.2***	6.3	6.3	6.2	6.2	6.2	6.2	0.0	**0.6**
butyl acetate	189	179	188	176	183	*183*	*5.6*	***3.0***	178.3	177.6	173.4	180.0	176.7	177.2	2.4	**1.4**
ethyl valerate	1.7	1.6	1.6	1.6	1.6	*1.6*	*0.1*	***3.7***	1.4	1.4	1.4	1.4	1.4	1.4	0.0	**0.9**
1,8-cineole	n.d.	n.d.	n.d.	n.d.	n.d.				0.008	0.010	0.011	0.009	0.007	0.009	0.001	**16.2**
ethyl capronate	647	595	604	592	599	*607*	*22.6*	***3.7***	528	530	523	525	525	526	2.8	**0.5**
hexyl acetate	36.0	32.7	33.0	32.9	32.9	*33.5*	*1.4*	***4.2***	28.3	28.3	28.1	28.0	28.0	28.1	0.1	**0.5**
ethyl heptanoate	1.9	1.8	1.8	1.8	1.8	*1.8*	*0.1*	***4.1***	1.5	1.5	1.5	1.5	1.5	1.5	0.0	**0.9**
cis rose oxide	5.5	5.0	5.0	5.0	5.0	*5.1*	*0.2*	***4.3***	5.1	5.2	5.1	5.0	5.1	5.1	0.1	**1.1**
trans-3-Hexen-1-ol	42.7	39.6	39.1	39.2	40.3	*40.2*	*1.5*	***3.7***	39.3	39.1	38.9	39.5	39.1	39.2	0.2	**0.6**
trans rose oxide	1.2	1.1	1.1	1.1	1.1	*1.1*	*0.0*	***4.5***	0.9	0.8	0.8	0.8	0.8	0.8	0.0	**1.3**
cis-3-Hexen-1-ol	19.6	18.1	18.0	18.1	18.3	*18.4*	*0.7*	***3.5***	18.0	17.7	17.7	18.1	17.9	17.9	0.2	**0.9**
ethyl caprylate	838	753	808	780	777	*791*	*32.6*	***4.1***	669	665	647	648	660	658	9.7	**1.5**
furfurylthiol	3.9	3.9	3.9	3.9	3.9	*3.9*	*0.0*	***0.3***	3.9	3.9	3.9	3.9	3.9	3.9	0.0	**0.3**
linalool oxide A	13.7	12.6	11.9	12.8	12.4	*12.7*	*0.7*	***5.4***	17.4	17.7	17.0	16.5	16.9	17.1	0.4	**2.5**
linalool oxide B	8.0	7.3	6.9	7.4	7.1	*7.4*	*0.4*	***5.6***	9.9	10.3	9.9	9.3	9.8	9.8	0.3	**3.5**
benzaldehyde	16.1	14.5	15.0	14.7	14.9	*15.0*	*0.6*	***4.2***	15.4	16.1	15.4	15.7	15.0	15.5	0.4	**2.6**
linalool	169	156	152	156	153	*157*	*6.9*	***4.4***	197	197	195	194	194	196	1.7	**0.9**
ethyl leucate	24.2	22.3	21.7	22.6	22.3	*22.6*	*1.0*	***4.2***	25.7	25.7	25.4	25.3	25.4	25.5	0.2	**0.7**
terpinen-4-ol	1.7	1.3	1.4	1.5	1.4	*1.5*	*0.1*	***9.2***	1.8	1.9	2.0	1.9	1.8	1.9	0.1	**3.8**
ethyl caprate	262	242	284	262	266	*263*	*14.9*	***5.6***	213	212	201	201	209	207	5.9	**2.9**
benzylmercaptan	2.4	2.4	2.4	2.4	2.4	*2.4*	*0.0*	***0.1***	2.4	2.4	2.4	2.4	2.4	2.4	0.0	**0.1**
phenylacetaldehyde	5.7	4.7	4.5	3.9	4.0	*4.6*	*0.7*	***15.8***	3.4	4.3	3.9	3.9	4.1	3.9	0.3	**8.3**
safranal	0.2	0.2	0.2	0.2	0.2	*0.2*	*0.0*	***4.0***	0.2	0.2	0.2	0.2	0.2	0.2	0.0	**2.6**
diethyl succinate	1158	1074	1104	1072	1068	*1095*	*37.7*	***3.4***	1085	1089	1060	1087	1085	1081	12.0	**1.1**
α-terpineol	53	49	48	49	48	*50*	*2.1*	***4.3***	66	66	65	64	65	66	0.8	**1.2**
valeric acid	25.1	23.7	24.6	22.8	24.1	*24.1*	*0.9*	***3.7***	23.6	23.0	24.5	24.0	23.9	23.8	0.5	**2.3**
β-citronellol	170	155	154	158	154	*158*	*6.6*	***4.2***	151	149	145	147	148	148	2.3	**1.6**
TDN	1.0	0.9	0.8	1.0	0.9	*0.9*	*0.1*	***8.0***	1.8	2.0	1.7	1.4	1.7	1.7	0.2	**13.1**

	Gewürztraminer A (40°C 4 days)								Gewürztraminer B (40°C 4 days)							
	**GW A1**	**GW A2**	**GW A3**	**GW A4**	**GW A5**				**GW B1**	**GW B2**	**GW B3**	**GW B4**	**GW B5**			
**Compounds**						***mean***	***Std dev.***	***cv%***						***mean***	***Std dev.***	***cv%***
nerol	86.2	79.4	77.6	81.5	76.8	80.3	3.8	4.7	65.1	63.5	64.8	61.8	62.9	63.6	1.4	2.1
ethyl phenylacetate	4.0	3.6	3.6	3.7	3.6	3.7	0.2	4.3	4.1	4.1	4.1	4.0	4.0	4.1	0.0	1.0
methyl salicylate	2.1	1.9	1.8	1.9	1.8	1.9	0.1	7.2	2.0	2.0	1.9	1.8	1.9	1.9	0.1	3.7
phenylethyl acetate	125	114	114	116	113	116	4.8	4.1	113	112	111	110	111	112	1.0	0.9
β-damascenone	3.6	3.2	3.2	3.2	3.2	3.3	0.2	5.7	4.4	4.4	4.2	4.2	4.4	4.3	0.1	2.3
ethyl laurate	8.0	7.9	10.1	9.1	9.2	8.9	0.9	10.5	9.1	8.2	8.5	9.2	8.3	8.7	0.5	5.3
geraniol	323	304	307	308	307	310	7.3	2.4	265	270	262	264	269	266	3.6	1.3
guaiacol	0.6	0.6	0.6	0.5	0.5	0.6	0.0	6.5	0.5	0.5	0.5	0.5	0.5	0.5	0.0	3.7
benzyl alcohol	104	95	96	95	95	97	3.8	3.9	95.9	94.2	93.3	95.2	94.2	94.6	1.0	1.1
trans-whiskey lactone	0.2	0.1	0.1	0.2	0.2	0.2	0.0	14.1	0.1	0.2	0.2	0.2	0.2	0.2	0.0	11.8
cis-whiskey lactone	0.4	0.3	0.3	0.3	0.3	0.3	0.0	11.6	0.4	0.3	0.3	0.4	0.3	0.3	0.0	12.3
4-ethyl guaiacol	0.2	0.2	0.2	0.2	0.2	0.2	0.01	3.1	0.2	0.2	0.2	0.2	0.2	0.2	0.0	4.1
γ-nonalactone	11.3	10.5	10.6	10.7	10.6	10.7	0.3	3.0	10.7	11.1	10.9	11.1	11.0	11.0	0.1	1.3
octanoic acid	4415	4011	4301	3966	4021	4143	201.6	4.9	4005	4036	3819	4042	4012	3983	93.0	2.3
ethyl cinnamate	1.7	1.6	1.6	1.6	1.6	1.6	0.1	3.8	1.6	1.6	1.6	1.6	1.6	1.6	0.0	0.9
γ-decalactone	1.5	1.5	1.5	1.5	1.4	1.5	0.0	2.2	1.5	1.4	1.5	1.5	1.4	1.5	0.1	3.8
4-ethyl-phenol	0.5	0.5	0.5	0.5	0.5	0.5	0.0	6.2	0.5	0.5	0.5	0.5	0.5	0.5	0.0	4.5
eugenol	8.6	8.0	7.6	8.1	7.9	8.0	0.3	4.2	8.0	7.9	7.8	7.7	8.0	7.9	0.1	1.6
4-vinylguaiacol	1017	951	957	962	947	967	28.6	3.0	912	906	896	896	905	903	7.0	0.8
δ-decalactone	7.4	6.4	6.3	6.6	6.3	6.6	0.4	6.7	8.2	8.4	7.9	7.5	8.4	8.1	0.4	4.7
2-aminoacetophenone	0.1	0.1	0.1	0.1	0.1	0.1	0.01	8.0	0.1	0.1	0.1	0.1	0.1	0.1	0.0	7.2
decanoic acid	2001	1773	1862	1888	1860	1877	82.0	4.4	1901	1850	1847	1875	1889	1872	23.6	1.3
geranic acid	804	740	739	749	733	753	29.1	3.9	760	739	744	730	746	744	11.0	1.5
γ-dodecalactone	21.1	20.0	18.5	15.7	20.5	19.2	2.2	11.2	19.9	19.6	19.3	14.0	19.4	18.4	2.5	13.4
zingerone	24.5	22.2	21.9	22.6	22.6	22.8	1.0	4.6	22.1	21.8	21.8	22.5	21.8	22.0	0.3	1.4

Table S4. Descriptive statistics of the measured volatile compounds and one-way Anova analysis and post-hoc test results for the Gewürztraminer wines.

Compound	Condition	Number of samples	Mean (µg/L)	Std. Deviation (µg/L)	F	Significance	Post Hoc Tests*
isobutyl acetate	control (t0)	7	46.15	9.79	13.06	<.001	a
	2.5 weeks (t1)	7	30.83	6.70			b
	5 weeks (t2)	7	24.85	7.30			b
ethyl butyrate	control (t0)	7	393.66	73.50	0.07	0.93	a
	2.5 weeks (t1)	7	402.39	43.76			a
	5 weeks (t2)	7	391.90	47.97			a
ethyl 2-methylbutyrate	control (t0)	7	9.77	4.74	6.22	0.01	a
	2.5 weeks (t1)	7	21.22	9.28			b
	5 weeks (t2)	7	26.20	11.44			b
ethyl isovalerate	control (t0)	7	19.15	10.36	6.01	0.01	a
	2.5 weeks (t1)	7	43.71	20.19			b
	5 weeks (t2)	7	54.00	24.58			b
butyl acetate	control (t0)	7	2.56	1.66	4.78	0.02	a
	2.5 weeks (t1)	7	1.33	0.77			ab
	5 weeks (t2)	7	0.82	0.37			b
ethyl valerate	control (t0)	7	1.64	0.40	0.39	0.68	a
	2.5 weeks (t1)	7	1.81	0.42			a
	5 weeks (t2)	7	1.79	0.36			a
1,8-cineole	control (t0)	7	0.04	0.02	15.83	<.001	a
	2.5 weeks (t1)	7	2.20	1.10			b
	5 weeks (t2)	7	3.57	1.73			b
ethyl capronate	control (t0)	7	791.56	135.67	6.51	0.01	a
	2.5 weeks (t1)	7	627.62	81.73			b
	5 weeks (t2)	7	592.29	106.82			b
ethyl capronate	control (t0)	7	780.97	153.35	4.66	0.02	a
	2.5 weeks (t1)	7	625.96	97.12			ab
	5 weeks (t2)	7	585.61	122.52			a
hexyl acetate	control (t0)	7	71.55	46.09	8.15	0.00	a
	2.5 weeks (t1)	7	26.30	13.01			b
	5 weeks (t2)	7	14.09	8.28			b
ethyl heptanoate	control (t0)	7	1.20	0.35	5.64	0.01	a
	2.5 weeks (t1)	7	0.85	0.26			b
	5 weeks (t2)	7	0.75	0.17			b
1-hexanol	control (t0)	7	1335.66	232.54	0.12	0.89	a
	2.5 weeks (t1)	7	1394.51	312.85			a
	5 weeks (t2)	7	1406.93	322.72			a
cis rose oxide	control (t0)	7	4.20	1.38	1.95	0.17	a
	2.5 weeks (t1)	7	3.38	1.32			a
	5 weeks (t2)	7	2.87	1.11			a
trans-3-hexen-1-ol	control (t0)	7	73.41	34.06	0.18	0.83	a
	2.5 weeks (t1)	7	67.15	27.67			a
	5 weeks (t2)	7	63.88	27.25			a
trans rose oxide	control (t0)	7	0.60	0.30	5.77	0.01	a
	2.5 weeks (t1)	7	0.32	0.11			b
	5 weeks (t2)	7	0.28	0.09			b
cis-3-hexen-1-ol	control (t0)	7	33.87	19.17	0.08	0.92	a
	2.5 weeks (t1)	7	31.11	17.12			a
	5 weeks (t2)	7	30.15	16.81			a
ethyl caprylate	control (t0)	7	821.61	152.47	51.41	<.001	a
	2.5 weeks (t1)	7	346.53	56.29			b
	5 weeks (t2)	7	305.86	84.70			b
linalool oxide A	control (t0)	7	17.34	4.35	65.47	<.001	a
	2.5 weeks (t1)	7	171.92	35.63			b
	5 weeks (t2)	7	228.77	50.51			c
linalool oxide B	control (t0)	7	8.89	1.78	69.47	<.001	a
	2.5 weeks (t1)	7	102.64	21.24			b
	5 weeks (t2)	7	135.69	29.21			c
benzaldehyde	control (t0)	7	3.67	2.06	6.76	0.01	a
	2.5 weeks (t1)	7	8.32	3.90			b
	5 weeks (t2)	7	10.91	4.73			b
linalool	control (t0)	7	214.83	110.40	11.58	<.001	a

Compound	Condition	Number of samples	Mean (µg/L)	Std. Deviation (µg/L)	F	Significance	Post Hoc Tests*
	2.5 weeks (t1)	7	84.68	37.73			b
	5 weeks (t2)	7	44.59	27.41			b
ethyl leuc ate	control (t0)	7	50.97	15.77	8.94	0.00	a
	2.5 weeks (t1)	7	88.02	23.54			b
	5 weeks (t2)	7	99.97	27.03			b
terpinen-4-ol	control (t0)	7	2.47	0.60	34.02	<.001	a
	2.5 weeks (t1)	7	8.28	1.61			b
	5 weeks (t2)	7	7.40	1.76			b
ethyl caprate	control (t0)	7	171.99	57.81	38.79	<.001	a
	2.5 weeks (t1)	7	35.04	13.16			b
	5 weeks (t2)	7	25.63	10.87			b
phenylacetaldehyde	control (t0)	7	19.62	5.00	7.43	0.00	a
	2.5 weeks (t1)	7	17.00	3.32			b
	5 weeks (t2)	7	32.38	12.46			b
safranal	control (t0)	7	0.14	0.01	126.53	<.001	a
	2.5 weeks (t1)	7	0.89	0.13			b
	5 weeks (t2)	7	1.13	0.16			c
diethyl succinate	control (t0)	7	3601.42	1693.79	17.08	<.001	a
	2.5 weeks (t1)	7	6777.60	1658.75			b
	5 weeks (t2)	7	9009.75	1860.68			c
α-terpineol	control (t0)	7	90.14	36.59	20.13	<.001	a
	2.5 weeks (t1)	7	319.94	90.05			b
	5 weeks (t2)	7	294.31	84.25			b
valeric acid	control (t0)	7	24.44	4.34	0.66	0.53	a
	2.5 weeks (t1)	7	24.35	2.67			a
	5 weeks (t2)	7	22.55	3.11			a
β-citronellol	control (t0)	7	69.38	31.32	12.44	<.001	a
	2.5 weeks (t1)	7	25.49	17.25			b
	5 weeks (t2)	7	15.47	10.45			b
TDN	control (t0)	7	0.87	0.30	25.51	<.001	a
	2.5 weeks (t1)	7	13.19	4.75			b
	5 weeks (t2)	7	13.35	4.42			b
ethyl phenylacetate	control (t0)	7	7.41	1.90	14.97	<.001	a
	2.5 weeks (t1)	7	13.71	3.34			b
	5 weeks (t2)	7	16.37	3.87			b
methyl salicylate	control (t0)	7	1.15	0.46	3.70	0.05	a
	2.5 weeks (t1)	7	1.86	0.61			a
	5 weeks (t2)	7	1.74	0.48			a
nerol	control (t0)	7	66.66	65.18	6.48	0.01	a
	2.5 weeks (t1)	7	6.11	5.29			b
	5 weeks (t2)	7	1.52	2.60			b
phenylethyl acetate	control (t0)	7	221.58	75.64	15.76	<.001	a
	2.5 weeks (t1)	7	114.09	32.53			b
	5 weeks (t2)	7	80.15	22.05			b
β-damascenone	control (t0)	7	2.39	0.54	4.60	0.02	a
	2.5 weeks (t1)	7	3.32	0.89			ab
	5 weeks (t2)	7	3.56	0.81			b
ethyl laurate	control (t0)	7	2.50	1.81	13.35	<.001	a
	2.5 weeks (t1)	7	n.d.[#]				b
	5 weeks (t2)	7	n.d.[#]				b
geraniol	control (t0)	7	166.18	138.86	8.17	0.00	a
	2.5 weeks (t1)	7	20.40	9.46			b
	5 weeks (t2)	7	11.23	6.90			b
guaiacol	control (t0)	7	0.92	0.49	6.37	0.01	a
	2.5 weeks (t1)	7	1.99	0.58			b
	5 weeks (t2)	7	2.43	1.19			b
benzyl alcohol	control (t0)	7	127.25	43.31	0.25	0.78	a
	2.5 weeks (t1)	7	113.89	33.86			a
	5 weeks (t2)	7	116.98	32.25			a
trans-whiskey lactone	control (t0)	7	0.34	0.40	4.99	0.02	a
	2.5 weeks (t1)	7	n.d.[#]				b
	5 weeks (t2)	7	n.d.[#]				b
γ-octalactone	control (t0)	7	1.68	0.75	14.55	<.001	a
	2.5 weeks (t1)	7	3.96	1.25			b
	5 weeks (t2)	7	1.29	0.93			b

Compound	Condition	Number of samples	Mean (µg/L)	Std. Deviation (µg/L)	F	Significance	Post Hoc Tests*
β-ionone	control (t0)	7	0.01	0.01	0.50	0.61	a
	2.5 weeks (t1)	7	0.01	0.02			a
	5 weeks (t2)	7	n.d.[#]				a
cis-whiskey lactone	control (t0)	7	0.61	0.61	1.51	0.25	a
	2.5 weeks (t1)	7	0.32	0.55			a
	5 weeks (t2)	7	1.86	2.93			a
benzothiazole	control (t0)	7	0.22	0.17	12.05	<.001	a
	2.5 weeks (t1)	7	0.72	0.14			b
	5 weeks (t2)	7	0.85	0.38			b
4-ethyl guaiacol	control (t0)	7	0.28	0.13	1.16	0.34	a
	2.5 weeks (t1)	7	0.30	0.13			a
	5 weeks (t2)	7	0.39	0.14			a
γ-nonalactone	control (t0)	7	8.79	4.35	0.26	0.77	a
	2.5 weeks (t1)	7	8.29	4.04			a
	5 weeks (t2)	7	9.93	4.66			a
octanoic acid	control (t0)	7	7576.39	1572.40	0.85	0.44	a
	2.5 weeks (t1)	7	8691.09	1856.72			a
	5 weeks (t2)	7	7807.36	1618.77			a
ethyl cinnamate	control (t0)	7	0.90	0.26	2.41	0.12	a
	2.5 weeks (t1)	7	0.68	0.20			a
	5 weeks (t2)	7	0.68	0.17			a
nonanoic acid	control (t0)	7	49.34	13.25	25.21	<.001	a
	2.5 weeks (t1)	7	87.77	14.45			b
	5 weeks (t2)	7	93.16	9.56			b
γ-decalactone	control (t0)	7	1.63	0.46	0.03	0.97	a
	2.5 weeks (t1)	7	1.65	0.40			a
	5 weeks (t2)	7	1.59	0.35			a
4-ethyl-phenol	control (t0)	7	0.39	0.24	2.33	0.13	a
	2.5 weeks (t1)	7	0.64	0.26			a
	5 weeks (t2)	7	0.68	0.31			a
eugenol	control (t0)	7	5.28	1.38	0.92	0.42	a
	2.5 weeks (t1)	7	6.07	1.23			a
	5 weeks (t2)	7	6.12	1.29			a
4-vinylguaiacol	control (t0)	7	700.24	231.39	16.18	<.001	a
	2.5 weeks (t1)	7	329.56	64.24			b
	5 weeks (t2)	7	307.97	73.73			b
δ-decalactone	control (t0)	7	7.95	1.73	23.59	<.001	a
	2.5 weeks (t1)	7	18.66	3.53			b
	5 weeks (t2)	7	17.79	4.01			b
2-aminoacetophenone	control (t0)	7	0.23	0.04	9.29	0.00	a
	2.5 weeks (t1)	7	0.43	0.12			ab
	5 weeks (t2)	7	0.62	0.26			b
decanoic acid	control (t0)	7	1978.96	743.38	0.22	0.81	a
	2.5 weeks (t1)	7	2212.56	885.89			a
	5 weeks (t2)	7	1964.76	727.51			a
geranic acid	control (t0)	7	352.73	136.06	1.46	0.26	a
	2.5 weeks (t1)	7	451.49	89.15			a
	5 weeks (t2)	7	397.14	94.10			a
γ-dodecalactone	control (t0)	7	51.93	39.39	0.46	0.64	a
	2.5 weeks (t1)	7	37.64	33.87			a
	5 weeks (t2)	7	37.36	22.42			a
zingerone	control (t0)	7	24.37	6.21	0.73	0.50	a
	2.5 weeks (t1)	7	27.99	6.12			a
	5 weeks (t2)	7	27.79	6.61			a

*Means within conditions that do not share a letter are significantly different (significance value below 0,05). #n.d: not detected (was considered zero for the Anova).

Table S5. Descriptive statistics of the measured volatile compounds and one-way Anova analysis results for **the** Teroldego wines.

Compound	Condition	Number of samples	Mean (µg/L)	Std. Deviation (µg/L)	F	Significance	Post Hoc Tests*
isobutyl acetate	control (t0)	7	57.09	2.93	0.12	0.89	a
	2.5 weeks (t1)	7	56.44	6.87			a
	5 weeks (t2)	7	58.07	8.20			a
ethyl butyrate	control (t0)	7	182.22	38.92	0.31	0.74	a
	2.5 weeks (t1)	7	191.12	42.55			a
	5 weeks (t2)	7	199.58	43.03			a
ethyl 2-methylbutyrate	control (t0)	7	13.41	5.49	6.23	0.01	a
	2.5 weeks (t1)	7	23.06	8.94			ab
	5 weeks (t2)	7	29.99	11.12			b
ethyl isovalerate	control (t0)	7	21.66	6.74	10.89	<.001	a
	2.5 weeks (t1)	7	38.28	11.43			b
	5 weeks (t2)	7	49.74	14.43			b
butyl acetate	control (t0)	7	1.43	0.38	0.25	0.78	a
	2.5 weeks (t1)	7	1.27	0.46			a
	5 weeks (t2)	7	1.29	0.51			a
isopentyl acetate	control (t0)	7	931.22	234.89	13.97	<.001	a
	2.5 weeks (t1)	7	606.01	99.56			b
	5 weeks (t2)	7	526.56	63.45			b
ethyl valerate	control (t0)	7	1.41	0.92	0.02	0.98	a
	2.5 weeks (t1)	7	1.38	0.89			a
	5 weeks (t2)	7	1.48	0.91			a
1,8-cineole	control (t0)	7	0.02	0.02	22.91	<.001	a
	2.5 weeks (t1)	7	0.05	0.02			b
	5 weeks (t2)	7	0.09	0.02			c
ethyl capronate	control (t0)	7	258.90	50.06	7.33	0.01	a
	2.5 weeks (t1)	7	192.08	29.14			b
	5 weeks (t2)	7	195.91	26.06			b
hexyl acetate	control (t0)	7	10.46	6.01	4.02	0.04	a
	2.5 weeks (t1)	7	6.96	3.68			ab
	5 weeks (t2)	7	4.13	1.69			b
ethyl heptanoate	control (t0)	7	0.80	0.27	7.30	0.01	a
	2.5 weeks (t1)	7	0.47	0.15			b
	5 weeks (t2)	7	0.47	0.13			b
trans-3-Hexen-1-ol	control (t0)	7	36.41	13.04	0.24	0.79	a
	2.5 weeks (t1)	7	33.13	11.51			a
	5 weeks (t2)	7	32.27	11.04			a
cis-3-Hexen-1-ol	control (t0)	7	145.56	52.57	0.30	0.74	a
	2.5 weeks (t1)	7	130.04	42.82			a
	5 weeks (t2)	7	128.07	41.85			a
ethyl caprylate	control (t0)	7	267.43	63.98	50.58	<.001	a
	2.5 weeks (t1)	7	89.76	14.10			b
	5 weeks (t2)	7	85.23	13.81			b
linalool oxide A	control (t0)	7	4.18	2.98	11.22	<.001	a
	2.5 weeks (t1)	7	11.73	4.65			b
	5 weeks (t2)	7	14.65	4.91			b
linalool oxide B	control (t0)	7	2.33	1.65	12.04	<.001	a
	2.5 weeks (t1)	7	6.78	2.64			b
	5 weeks (t2)	7	8.43	2.77			b
benzaldehyde	control (t0)	7	11.51	8.36	0.09	0.92	a
	2.5 weeks (t1)	7	13.66	11.58			a
	5 weeks (t2)	7	13.45	11.21			a
linalool	control (t0)	7	11.88	1.40	99.44	<.001	a
	2.5 weeks (t1)	7	4.66	1.41			b
	5 weeks (t2)	7	2.94	0.90			c
ethyl leuc ate	control (t0)	7	99.79	27.80	2.71	0.09	a
	2.5 weeks (t1)	7	126.16	27.80			a
	5 weeks (t2)	7	133.57	29.93			a
terpinen-4-ol	control (t0)	7	0.67	0.59	0.06	0.94	a
	2.5 weeks (t1)	7	0.64	0.45			a
	5 weeks (t2)	7	0.58	0.34			a

Compound	Condition	Number of samples	Mean (µg/L)	Std. Deviation (µg/L)	F	Significance	Post Hoc Tests*
ethyl caprate	control (t0)	7	57.14	32.92	16.66	<.001	a
	2.5 weeks (t1)	7	6.34	3.96			b
	5 weeks (t2)	7	5.27	2.81			b
phenylacetaldehyde	control (t0)	7	30.42	5.29	4.24	0.03	a
	2.5 weeks (t1)	7	47.35	15.69			ab
	5 weeks (t2)	7	43.23	10.57			b
safranal	control (t0)	7	0.14	0.01	268.33	<.001	a
	2.5 weeks (t1)	7	0.77	0.07			b
	5 weeks (t2)	7	0.99	0.10			c
α-terpineol	control (t0)	7	4.87	1.22	70.47	<.001	a
	2.5 weeks (t1)	7	12.32	1.27			b
	5 weeks (t2)	7	12.41	1.58			b
valeric acid	control (t0)	7	18.05	6.81	0.14	0.87	a
	2.5 weeks (t1)	7	20.32	8.74			a
	5 weeks (t2)	7	19.03	8.36			a
β-citronellol	control (t0)	7	16.69	4.75	22.98	<.001	a
	2.5 weeks (t1)	7	8.74	2.55			b
	5 weeks (t2)	7	4.95	1.93			b
TDN	control (t0)	7	0.36	0.18	27.84	<.001	a
	2.5 weeks (t1)	7	4.37	1.82			b
	5 weeks (t2)	7	5.84	1.64			b
ethyl phenylacetate	control (t0)	7	9.24	2.56	6.81	0.01	a
	2.5 weeks (t1)	7	13.55	3.56			ab
	5 weeks (t2)	7	16.18	4.31			b
methyl salicylate	control (t0)	7	3.25	1.65	0.03	0.97	a
	2.5 weeks (t1)	7	3.46	1.64			a
	5 weeks (t2)	7	3.43	1.65			a
nerol	control (t0)	7	4.34	3.32	12.00	<.001	a
	2.5 weeks (t1)	7	n.d.#				b
	5 weeks (t2)	7	n.d.#				b
phenylethyl acetate	control (t0)	7	119.77	49.75	3.06	0.07	a
	2.5 weeks (t1)	7	84.76	27.76			a
	5 weeks (t2)	7	75.66	21.76			a
β-damascenone	control (t0)	7	2.41	0.41	8.57	0.00	a
	2.5 weeks (t1)	7	3.52	0.73			b
	5 weeks (t2)	7	3.67	0.68			b
ethyl laurate	control (t0)	7	1.37	0.60	35.80	<.001	a
	2.5 weeks (t1)	7	n.d.#				b
	5 weeks (t2)	7	n.d.#				b
geraniol	control (t0)	7	6.88	2.57	28.78	<.001	a
	2.5 weeks (t1)	7	1.82	0.64			b
	5 weeks (t2)	7	0.95	0.68			b
guaiacol	control (t0)	7	5.21	2.24	30.67	<.001	a
	2.5 weeks (t1)	7	19.77	4.99			b
	5 weeks (t2)	7	25.32	6.63			b
benzyl alcohol	control (t0)	7	218.86	113.95	0.02	0.98	a
	2.5 weeks (t1)	7	207.95	106.24			a
	5 weeks (t2)	7	216.21	108.05			a
trans-whiskey lactone	control (t0)	7	6.59	8.33	0.00	1.00	a
	2.5 weeks (t1)	7	6.50	8.24			a
	5 weeks (t2)	7	6.73	8.50			a
γ-octalactone	control (t0)	7	0.76	0.16	2.54	0.11	a
	2.5 weeks (t1)	7	1.00	0.25			a
	5 weeks (t2)	7	0.96	0.21			a
β-ionone	control (t0)	7	0.13	0.05	2.57	0.10	a
	2.5 weeks (t1)	7	0.10	0.03			a
	5 weeks (t2)	7	0.09	0.02			a
cis-whiskey lactone	control (t0)	7	14.26	20.59	0.00	1.00	a
	2.5 weeks (t1)	7	14.12	20.28			a
	5 weeks (t2)	7	14.47	20.91			a
benzothiazole	control (t0)	7	0.48	0.52	1.88	0.18	a
	2.5 weeks (t1)	7	0.96	0.52			a
	5 weeks (t2)	7	0.91	0.52			a
4-ethyl guaiacol	control (t0)	7	2.42	3.63	0.00	1.00	a
	2.5 weeks (t1)	7	2.53	3.78			a

Compound	Condition	Number of samples	Mean (µg/L)	Std. Deviation (µg/L)	F	Significance	Post Hoc Tests*
	5 weeks (t2)	7	2.60	3.87			a
γ-nonalactone	control (t0)	7	8.49	3.67	0.37	0.70	a
	2.5 weeks (t1)	7	9.61	4.09			a
	5 weeks (t2)	7	10.34	4.41			a
octanoic acid	control (t0)	7	2311.98	490.36	1.20	0.32	a
	2.5 weeks (t1)	7	2697.27	463.49			a
	5 weeks (t2)	7	2446.87	460.68			a
ethyl cinnamate	control (t0)	7	0.94	0.49	0.48	0.63	a
	2.5 weeks (t1)	7	0.72	0.43			a
	5 weeks (t2)	7	0.77	0.41			a
nonanoic acid	control (t0)	7	85.52	15.14	3.01	0.08	a
	2.5 weeks (t1)	7	102.42	15.33			a
	5 weeks (t2)	7	101.30	12.61			a
γ-decalactone	control (t0)	7	0.79	0.29	0.11	0.89	a
	2.5 weeks (t1)	7	0.74	0.22			a
	5 weeks (t2)	7	0.73	0.25			a
4-ethyl-phenol	control (t0)	7	35.76	69.53	0.00	1.00	a
	2.5 weeks (t1)	7	38.56	74.95			a
	5 weeks (t2)	7	37.10	72.00			a
eugenol	control (t0)	7	5.67	3.03	0.11	0.90	a
	2.5 weeks (t1)	7	6.28	3.22			a
	5 weeks (t2)	7	6.41	3.25			a
4-vinylguaiacol	control (t0)	7	7.30	2.08	29.44	<.001	a
	2.5 weeks (t1)	7	12.91	2.62			b
	5 weeks (t2)	7	17.16	2.49			c
δ-decalactone	control (t0)	7	6.79	1.04	4.24	0.03	a
	2.5 weeks (t1)	7	8.35	1.98			ab
	5 weeks (t2)	7	9.55	2.12			b
2-aminoacetophenone	control (t0)	7	0.27	0.06	7.24	0.01	a
	2.5 weeks (t1)	7	0.22	0.01			b
	5 weeks (t2)	7	0.19	0.03			b
decanoic acid	control (t0)	7	701.71	304.99	0.08	0.93	a
	2.5 weeks (t1)	7	701.40	304.63			a
	5 weeks (t2)	7	648.00	270.72			a
geranic acid	control (t0)	7	34.98	4.22	3.94	0.04	a
	2.5 weeks (t1)	7	39.45	4.39			ab
	5 weeks (t2)	7	33.56	3.64			b
γ-dodecalactone	control (t0)	7	29.43	6.61	7.69	0.00	a
	2.5 weeks (t1)	7	18.18	5.00			b
	5 weeks (t2)	7	19.67	5.77			b
	control (t0)	7	1.03	0.70	0.23	0.80	a
zingerone	2.5 weeks (t1)	7	0.84	0.47			a
	5 weeks (t2)	7	0.88	0.46			a

*Means within conditions that do not share a letter are significantly different (significance value below 0,05). #n.d: not detected (was considered zero for the Anova).

Table S6: basic enological analysis of commercial wines used for accelerating aging

Wine	Cultivar	pH	Total acidity (g/L)	Volatile acidity (g/L)	Alcool (%)	Sugars (g/L)
**G1**	**Gewürztraminer**	3.4	6.17	0.3	14.68	8.84
**G2**	**Gewürztraminer**	3.7	5.27	0.41	15.27	7.52
**G3**	**Gewürztraminer**	3.7	5.43	0.22	14	4.3
**G4**	**Gewürztraminer**	3.6	5.64	0.51	14.63	4.43
**G5**	**Gewürztraminer**	3.7	5.95	0.18	14.33	6.38
**G6**	**Gewürztraminer**	3.9	5.77	0.2	15.42	9.43
**G7**	**Gewürztraminer**	3.7	5.21	0.21	15.08	5.55
**T1**	**Teroldego**	3.9	5.63	0.3	12.56	2.9
**T2**	**Teroldego**	4.0	5.37	0.36	12.71	4.64
**T3**	**Teroldego**	3.9	5.59	0.33	13.47	2.59
**T4**	**Teroldego**	3.9	5.64	0.38	12.82	2.12
**T5**	**Teroldego**	4.1	5.14	0.57	13.06	3.1
**T6**	**Teroldego**	3.8	6.18	0.38	13.17	0.51
**T7**	**Teroldego**	3.9	5.44	0.39	13.34	0.76
